# Detection, communication, and individual identification with deep audio embeddings: A case study with North Atlantic right whales

**DOI:** 10.1371/journal.pcbi.1013321

**Published:** 2026-06-30

**Authors:** Irina Tolkova, Holger Klinck, Dana A. Cusano, Anke Kügler, Susan E. Parks

**Affiliations:** 1 Cornell K. Lisa Yang Center for Conservation Bioacoustics, Cornell University, Ithaca, New York, United States of America; 2 Marine Mammal Institute, Department of Fisheries, Wildlife, and Conservation Sciences, Oregon State University, Newport, Oregon, United States of America; 3 Department of Biology, Syracuse University, Syracuse, New York, United States of America; Leiden University: Universiteit Leiden, NETHERLANDS, KINGDOM OF THE

## Abstract

Anthropogenic noise has increased ambient sound levels across the globe, both underwater and on land. Among its many negative impacts, heightened noise can impair communication in vocal animals through acoustic masking. Conceptually, noise reduces the animal’s *communication space* – the area in which an individual animal can effectively convey information to a conspecific listener. Previous studies have estimated the communication space using sound propagation models and/or behavioral studies. However, studies frequently equate signal recognition with signal detection – a necessary but not sufficient precondition – thereby persistently overestimating spatial coverage and underestimating anthropogenic impacts. Measuring communication is inherently difficult, and varies with taxa, call type, and context, leading to significant data gaps in key parameters. We propose that deep learning creates an opportunity to estimate biologically-relevant communication, even for data-limited species. In particular, we present a case study with the critically endangered North Atlantic right whale (*Eubalaena glacialis*; hereafter NARW). Prior research has demonstrated that the upcall – a low-frequency contact call produced across ages and sexes – encodes individual identity. We therefore consider a dataset of NARW vocalizations recorded with on-animal archival tags, spanning 234 samples across 11 individuals from 3 sites. First, we demonstrate that audio embeddings from the BirdNET model can robustly distinguish individual right whales. Then, we simulate the effect of varying ambient noise levels to estimate signal excess for both signal detection and individual identification, finding that an additional ≥7 dB is necessary for the model to distinguish individuals. Altogether, we hope this work provides both a methodological advance for individual identification and a framework for better understanding anthropogenic impacts on vocal wildlife.

## Author summary

Noise pollution from traffic, construction, and industry has become widespread not only on land but also throughout the world’s oceans. Beyond the health impacts of chronic noise exposure on people and wildlife, noise also interferes with animals’ essential need to communicate with each other. This impact is particularly concerning for marine mammals, for whom sound is crucial for social communication, navigation, finding food, and other key aspects of life. Yet accurately quantifying these impacts can be difficult. Typically, studies consider the space in which one animal would be able to detect a call from another, and evaluate how this area decreases with increasing noise levels. However, being able to *detect* a sound is not the same as being able to *communicate*. In this work, we aim to improve upon prior methods for estimating noise impacts by using emerging deep learning techniques to quantify a particular aspect of communication – recognizing which individual is calling. Specifically, we perform a case study for the critically endangered North Atlantic right whale using recordings from on-animal tags. We confirm that deep learning can be used to distinguish individual right whales, and that individual identification requires higher sound quality than simple detection. The proposed framework can be applied for other species and environments to improve assessments of noise impacts on wildlife.

## Introduction

Since the industrial revolution, anthropogenic noise has vastly reshaped global soundscapes. On land, transportation noise from automobiles and airplanes has given rise to elevated noise levels even in remote natural areas [1]. The oceans have experienced even greater change, due to exponential expansion of commercial shipping, oil and gas exploration, military sonar, underwater construction, and other sources [2,3]. In humans, chronic noise exposure has been associated with adverse health impacts ranging from increased risk of cardiovascular disease [4,5] to cognitive impairment [6,7]. In animals, studies have documented wide-ranging physiological and behavioral responses to noise [8,9]. Marine mammals are particularly vulnerable to noise impacts: most rely heavily on sound for key life functions from foraging to navigation, while living in an environment where anthropogenic noise is extensive, propagates efficiently to great distances, but may not be immediately evident to the public and policymakers. Alongside effects on physiological health, increased ambient noise can mask vocalizations, impacting the ability of animals to communicate. Consequently, changes in vocal activity have been observed across taxa in response to increased noise, ranging from increasing call amplitude (known as the Lombard effect) [10–12], increasing call frequency (thereby reducing masking by low-frequency noise sources) [13,14], or changing call rate and duration [15,16].

By obscuring a signal with noise, acoustic masking decreases an animal’s ability to communicate. Conceptually, masking reduces the animal’s “communication space” – the space in which the animal can effectively perceive and convey vocal information. Early studies aimed to quantify this region by measuring behavioral changes in response to call playback at different signal-to-noise ratios (SNR) [17–19]. While these studies yield numerical thresholds for a visible response, they fall short of truly measuring communication. Is the meaning behind the vocalizations still transmitted at the specified SNR thresholds, or are the animals simply detecting familiar sounds, too distant and faint to be understood?

Lohr et al [20] shed some light on this question through a laboratory study of budgerigars and zebra finches trained to indicate both detection and discrimination of conspecific and hetero-specific calls. The authors demonstrated that call discrimination was indeed more challenging than detection, requiring over 3 dB higher SNR on average. Integrated with a simple model of acoustic propagation, this difference would imply a discrimination distance under 70% of the detection distance, and consequently a 2-dimensional discrimination space about half the size of the detection space. Building on these findings, Dooling et al [21] suggested a conceptual framework with further tiered levels of communication: (1) on the outside, the detection space, in which an animal can detect a vocalization; (2) within it, the discrimination space, in which an animal can discriminate between multiple vocalizations; (3) next, the recognition space, in which an animal can recognize and interpret the vocalizations; (4) and finally, the innermost space of comfortable communication. In a basic propagation model assuming simple spherical spreading, these spaces can be imagined as concentric circles around the speaker or listener.

While proposed for birds, this framework can be translated across taxa, and has been considered for another group deeply impacted by anthropogenic noise: marine mammals [22]. Sound is the principal form of communication underwater – critical for social interaction, navigation, hunting and foraging, and other key life functions. In the United States, the Marine Mammal Protection Act, enacted in 1972, mandates the assessment and mitigation of adverse impacts on marine mammals – aptly including the impacts of acoustic masking [23]. Accordingly, significant study has been devoted to establishing scientifically-grounded species-specific thresholds at which noise affects communication [22]. However, properly defining communication is a challenging and highly context-dependent question. Behavioral studies as performed with laboratory birds may be too effort-intensive and prohibitively invasive, if not unethical, to conduct with marine mammals. Indeed, a large-scale review of masking in marine mammals highlights “Studies on signal excess required for signal detection versus discrimination, recognition and comfortable communication” as a research need, though noting: *The signal excess needed for comfortable communication is difficult (if not impossible) to measure in animals* [22].

Computationally, estimates of detection spaces are approached by formulating a sonar equation to calculate received sound levels, with appropriate terms for source levels, noise levels, and transmission loss, and modeling acoustic propagation to evaluate received SNR across an environment [22,24]. These studies recognize the need for an additional term to represent not just detection but information transfer; yet due to the lack of data, this term is either guessed or simply dropped. In consequence, the terms “active space,” “communication space,” and “detection space” are inconsistently defined in scientific literature, and frequently used interchangeably. This distinction is not just a conceptual concern, but carries practical consequences for conservation. As detecting a signal is a necessary but not sufficient condition for effective communication, studies that conflate the two will persistently over-estimate the communication space, and thereby under-estimate the adverse effects of anthropogenic noise in impact assessments.

We propose that machine learning can aid in bridging this gap. In the last decade, the field of bioacoustics has undergone a technological revolution from the widespread adoption of machine learning tools [25,26]. Deep neural networks have enabled automated detection and classification of diverse animal vocalizations within long audio recordings, greatly expanding the spatial and temporal scales of acoustic analysis. In particular, these models have shown very high sensitivity to acoustic characteristics across scales, identifying attributes of species, call types, regional dialects, and even individuals [27,28]. In this work, we aim to utilize deep learning to quantify an ecologically-meaningful form of communication and thereby estimate thresholds for detection and recognition. We consider a case study of a species acutely impacted by anthropogenic noise: the North Atlantic right whale (*Eubalaena glacialis*; hereafter NARW). First, we build on prior work to demonstrate that individual NARW can be distinguished through their vocalizations in an automated framework, without the need for manual feature selection. Second, we show that the ability to distinguish individuals declines as background noise levels increase, quantify this relationship as a function of the signal-to-noise ratio (SNR), and compare the sound levels required for detection and individual identification. Methodologically, we address a collection of challenges: leveraging deep learning for a small dataset, verifying robustness to noise, and adapting a model trained on higher-frequency audio to lower-frequency data. We then contextualize these results within prior findings, and discuss the implications of our analysis for improving estimates of anthropogenic impacts on a critically endangered species.

## Background

### Audio embeddings

While deep learning has revolutionized bioacoustic classification [26], sample sizes collected from tagging studies have typically been too small for training deep neural networks from scratch. Instead, networks trained on large-scale datasets may be successfully applied to new, previously unseen categories and tasks through transfer learning [29]. One form of transfer learning proposes that a neural network trained for an audio classification task will internally represent meaningful acoustic features: deeper layers will extract increasingly refined characteristics, converted into an output classification label by the final layer(s). In this framework, the activations of a penultimate layer of a neural network would be meaningful “feature vectors” specialized for the dataset. These feature vectors, commonly termed *embeddings*, can then be visualized, compared, or analyzed further with simple classification algorithms [26,30]. Transfer learning has proven to be an effective methodology for detection and classification of diverse sound types at low sample sizes – particularly valuable for monitoring rare and threatened species. These successes have motivated further interest in developing “foundation” models to represent acoustic characteristics across increasingly expansive datasets, and generalize across increasingly diverse bioacoustic tasks.

Consequently, a variety of models for calculating embeddings have emerged in recent years and this space continues to evolve rapidly [31,32]. Unlike study-specific models that focus on a single domain and task, foundation models are typically composed of millions to hundreds of millions of parameters [31], and differ widely in architecture, input and output representations, datasets, and training paradigms [31]. In particular, a key distinction lies between a supervised learning paradigm, in which models are trained for classification tasks, and self-supervised learning, in which models learn lower-dimensional representations of the input data without the need for expert labels. For instance, Perch [33] and BirdNET [34] are convolutional neural networks based on an EfficientNet backbone [35], trained on the supervised task of species-level bird call and song classification. In contrast, models such as AVES [36] and TweetyBERT [37] are transformer-based networks trained in a self-supervised framework, effectively learning optimized compression of input audio data. Most models operate on linear- or mel-scale spectrogram representations of short audio windows (typically 3–10 seconds), though some, such as AVES, operate directly on raw audio. The output size of embedding vectors also varies by model, from a few hundred to over one thousand dimensions [31]. Finally, other design choices include spectrogram parameters (such as window size and frequency range), training datasets (such as whether music or general audio is included), data augmentations (such as scaling, shifting, and masking), and the approach used for adapting models to downstream tasks [31].

Due to this broad variability in models, multiple studies have conducted comparative performance evaluations across a standardized set of downstream tasks. Kather et al [38] presented an analysis of 15 models for quality of feature extraction across two datasets, finding highest overall performance by BirdNET and Perch. In a different study, Miron et al [32] benchmark an array of models across 26 datasets to similarly find competitive performance by these two models, with BirdNET scoring highest across all models on the task of individual identification. In this study, we chose to work with BirdNET for its strong empirical performance, accessible user interface, wide practitioner adoption, and demonstrated robustness; however, the proposed methodology is model-agnostic and can be integrated with other bioacoustic foundation models.

BirdNET is trained on species-level classification of (primarily) bird vocalizations from repositories including Xeno-Canto, the Macaulay Library, and labeled global soundscapes [39]. Audio recordings are internally split into 3-second windows with user-specified overlap, transformed to spectrograms, passed through a series of EfficientNet-based Inverted Residual Blocks, followed by a global pooling operation to produce the embedding layer, which is then converted to species-level predictions with a final densely-connected layer [40]. It has been applied for transfer learning across diverse taxa, including marine mammals [41], primates [42,43], and frogs [44], as well as for varying tasks, such as classification of dialects and call types [27,39]. BirdNET is accessible through the graphical user interface BirdNET-Analyzer, via command-line, through libraries for Python and R, and other open-source tools [45].

### Neural networks and auditory perception

Our analysis leverages the properties of audio embeddings as a proxy for the perceptive capabilities of marine mammals. Clearly, this is an imperfect comparison, and one that is difficult to verify. Yet the heuristics currently used for estimating detection and recognition thresholds are also coarse, and often not specific to the scientific question, call type, or even species. For instance, a seminal work by Clark et al (2009) [24] suggested 10 dB as a detection threshold, and 16 dB as the detection-communication gap; values which were subsequently used in communication space studies for a variety of baleen whales, call types, and habitats [46–48]. However, the 10 dB value was a general estimate used across marine mammal studies [46], not accounting for high frequency dependence of this parameter [49]; the 16 dB value was derived from a rule-of-thumb estimate based on the duration-bandwidth product of the signal. The wide utilization of these imperfect estimates underscores the data deficiency in marine mammal perception, and thereby an opportunity for computational methods to help fill this gap – particularly in light of the strong parallels revealed by recent studies between deep neural networks (DNNs) and biological auditory processing [50,51].

DNNs have been shown to match and even outperform human participants across a wide range of challenging auditory tasks, galvanizing interest in parallels between artificial and biological neural processing [50,52,53]. Comprehensive studies have demonstrated remarkable convergence between DNNs and human brains in their underlying representations of language and vision [51]. In the auditory realm, activations across a span of DNNs showed strong correspondences to different brain regions indicated by functional Magnetic Resonance Imaging (fMRI) scans, suggesting that deep networks consistently learn similar pathways of underlying computational processing [54,55]. When compared against traditional models of the auditory cortex, DNNs have been shown to better predict human auditory response [54]. Neural networks have been used to understand specific principles of human auditory perception – such as the development of frequency-dependent interaural time and level differences for sound source localization [56] or the impacts of impairment on hearing in low-SNR conditions [57]. In sum, DNNs have become a valuable proxy for the study of human perception, offering answers not to just *how*, but even *why* human brains work as they do [58].

To be sure, human audition may not be representative of hearing in other animals. The biological mechanisms underlying hearing differ in the presence of the outer ear (pinnae), structure of the tympanic middle ear, audible frequency range, capacity for vocal learning, and other aspects [59,60]. Yet, key structural mechanisms such as the tympanic membrane, brainstem pathways, inner ear sensitivity, efferent feedback – and fundamental cognitive elements such as pitch perception, sound localization, and novelty detection, among others – are remarkably similar across vertebrates [61–64]. Notably, despite different mediums of propagation, studies have shown that humans and marine mammals have comparable capacity for discriminating frequency, intensity, and duration [65,66]. Moreover, the structure of deep neural networks is not specific to human anatomy; rather, human bias is introduced through anthropocentric training data. Evaluating the quality of the perceptual representation of DNNs for animals is innately challenging, though not unprecedented: Morfi et al (2021) have aimed to construct an acoustic embedding space which reflects perceptual similarity of chaffinch song based on experimental measurements [67]. Altogether, considering the demonstrated efficacy of DNNs for insights in neuroscience, the comparative similarities of auditory neural pathways, and the scarcity of data on acoustic perception in wild animals, we argue that DNNs fine-tuned on study-specific bioacoustic data offer a meaningful pathway for understanding and estimating animal communication.

### North Atlantic right whales

Specifically, we present a case study for a critically endangered species known to be gravely impacted by anthropogenic noise – the North Atlantic right whale (*Eubalaena glacialis*; hereafter NARW). NARW are slow-moving, surface-dwelling baleen whales that live along the North Atlantic coast. An easy target for harpoons, NARW were hunted to near-extinction by the start of the 20th century [68]. While NARW received protections from commercial whaling in 1935, their population struggled to recover, increasingly faced with different lethal threats: collisions with ships and entanglement in fishing gear [69]. Now numbering fewer than 400 individuals, NARW are a crucial focus in marine conservation [70].

While ship strike and entanglement are evident through scars, dragged gear, or necropsies, the effect of anthropogenic noise is understood to be severe but less visually apparent. Like all whales, NARW rely on sound for communication, vocalizing in a repertoire of low-frequency (< 500 Hz) calls [71]. Historically, this frequency range would have minimal interference from other sound sources, allowing these calls to propagate for many kilometers [72]. However, since 1950, commercial shipping has grown rapidly across the globe, with a ten-fold increase in gross tonnage by 2010 [2]. Correspondingly, measurements of ambient noise in the 25–50 Hz band have increased by approximately 3.3 dB per decade [2,73–75]. NARW have been shown to increase both amplitude and frequency of their calls in response to noise, which could reduce the effect of masking, but likely with an energetic cost [76]. Unsurprisingly, high noise levels have been linked to increased stress hormone production in NARW [77], affecting individual welfare and likelihood of reproduction.

Of the NARW repertoire, the most common call is the upcall: an upsweep from 50 to 350 Hz, 1–2 seconds in duration, that serves as a contact call for NARW of all ages and sexes [78]. While considered to be heavily stereotyped, an analysis of upcalls recorded on on-animal archival tags has shown that these vocalizations contain sufficient variability to distinguish individual whales with high accuracy [79]. Vocally distinguishing individual conspecifics may be highly consequential to a migratory and social species – particularly for mother-calf pairs to maintain contact throughout long-distance movement. Soon after birth, calves migrate north with their mothers, from the southeast U.S. coast up to Canadian waters [80]. While calves grow more independent over their first year of life, they are still nursing, and therefore dependent on their mothers for survival [81]. In fact, while mothers and calves typically separate within a year, studies in recent years have observed mothers and calves to remain together after weaning [82]. Acoustic masking is of particular concern, as mothers have been shown to communicate with lower-amplitude calls while raising young – likely to avoid drawing attention to their vulnerable calves [83], but consequently increasing the risk of losing each other amidst the noise. Past mother-calf pairs, individual identification may be significant for NARW social cohesion. NARW are highly social – commonly displaying play within surface-active groups (SAGs) [84] and maintaining non-random associations across years and regions [85]; these visual observations may yet underestimate social bonds sustained through acoustic communication [84]. Altogether, recordings of upcalls provide a unique opportunity to quantify one form of informational content – individual identity – and approximate the communication space of calling NARW under different noise regimes.

## Data collection

### Ethics statement

The original audio data collection was approved under Canadian Department of Fisheries and Oceans and United States National Marine Fisheries Service permits and Institutional Animal Care and Use Committees (IACUCs).

### Dataset construction

**Fig 1:**
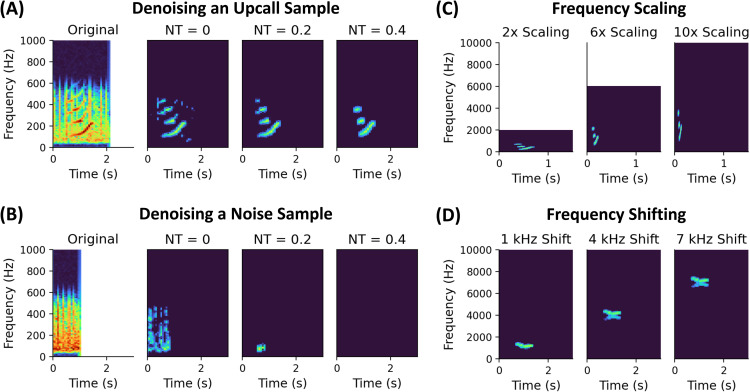
**(A)** The leftmost spectrogram displays an upcall sample with significant background noise. The following spectrograms display the sample after processing at different denoising thresholds, NT: 0, 0.1, and 0.2. **(B)** To de-couple classification of individual identity from classification of tag-specific noise, the same processing pipeline is applied to a corresponding noise sample for each upcall. **(C)** Frequency scaling with factors of 2x, 5x, and 10x is applied to a de-noised upcall sample. This approach compresses the duration and proportionally stretches the frequency range of the signal. **(D)** Frequency shifting at shifts of 1 kHz, 4 kHz, and 7 kHz is applied to a de-noised upcall sample. This approach maintains both the duration and difference between the maximum and minimum frequencies of the signal.

We leveraged a dataset of acoustic recordings from three key regions of North Atlantic right whale range [86]: the Bay of Fundy, Canada [87–89]; Cape Cod Bay, Massachusetts, USA [90]; and the Southeastern US [91]. Please reference McCordic et al (2016) [79] for thorough detail on data collection. After observation and photo-identification of all NARW in the field, audio recordings were collected with suction-cup-attached digital archival tags, including DTAGS [89] and Acousonde tags [92]. Upcalls were visually and aurally identified and labeled on spectrograms by expert observers using Raven Pro v.1.5. To ensure confidence in individual IDs, upcalls were screened for high signal-to-noise ratio and for time periods when tagged whales were observed to be alone during focal follows. Additionally, a 1-second ambient noise sample was annotated in the time period immediately before each upcall. We considered individuals with at least 10 upcall samples to avoid misleading accuracies over small sample sizes, resulting in a total of 234 upcalls across 11 individuals; the number of calls per individual varied from 10 to 54 with a median of 12. Each upcall was resampled to a 2kHz sampling rate, bandpass filtered to a range of 50–500 Hz with a Butterworth filter, normalized by the mean amplitude, and standardized to a length of 3 seconds by zero-padding. Examples of an upcall spectrogram are shown in Fig 1.

## Methods

Our methodology entails four key analytical steps. First, to construct a meaningful lower-dimensional representation of NARW upcalls, we use audio embeddings from the pre-trained deep neural network BirdNET and evaluate classification performance for individual identification. To ensure that noise is not a decisive confounding factor in our analysis, we apply denoising, and analyze a noise dataset in parallel with the upcall dataset. To optimize the performance of BirdNET for low-frequency whale calls, we apply and evaluate two forms of frequency modification. Lastly, after demonstrating the potential for individual identification, we vary SNR to quantify the impact of ambient noise levels on the accuracy of both detection and identification.

### Embeddings and evaluation metrics

To evaluate the potential for differentiating individual right whales with deep-learning-based acoustic features, we calculated BirdNET embeddings using the command-line interface for BirdNET Analyzer v1.5.1 [34] with model V2.3, yielding one vector of 1024 features for each 3-second audio sample. As all upcall samples were within a 3-second duration, this dataset did not require additional temporal manipulation or pooling. Then, to visualize the distribution of the embeddings, we applied UMAP [93] to reduce feature dimensionality from 1024 to 2, and visualized clustering structure within the distributions of embedded points. Next, we evaluated the performance of a simple classifier to distinguish individuals in the lower-dimensional embedding space. For greatest interpretability, we chose a linear support vector machine (SVM), which most directly quantifies linear separability of classes within the high-dimensional space of embeddings. The performance of the SVM was compared with a baseline random classifier, which always predicted the most common class observed in the training data. As the small dataset size rendered the division of the dataset into separate training and test sets impractical, we instead evaluated leave-one-out cross-validation (CV) accuracy, consistent with prior work [79].

### Denoising

One common pitfall in the analysis of data with deep learning methods is that models may learn and characterize aspects of a recording other than the desired target signal. As the recordings for an individual NARW were associated with a single tag in a particular environment, the upcalls were likely to contain individual-specific ambient noise profiles, creating the possibility that the BirdNET embeddings were representing characteristics of tag-specific noise rather than of individual-specific upcalls. To evaluate this effect, we constructed a “control” dataset of noise samples which was analyzed in parallel to the upcall dataset. Specifically, we selected 1-sec samples immediately before each upcall to represent the associated ambient noise profiles, and applied the BirdNET + SVM classification pipeline to this additional dataset. If classification performance was driven primarily by upcall characteristics, we would expect the analysis of noise data to yield little to no clustering and low cross-validation accuracies.

Indeed, preliminary analysis showed high classification accuracies across both the upcall and noise datasets. We accounted for this complication by applying rigorous denoising to both datasets. Specifically, we calculated spectrograms for both the upcall and noise samples with a Hann window of length 256 samples (0.128 seconds) with 50% overlap, and extracted the rows representing the 50–500 Hz range. To account for the spectral characteristics of the ambient noise, we subtracted the time-averaged spectrum of the noise spectrogram from the magnitudes of each column of the associated call spectrogram. Similarly, to address impulsive or narrowband noises, we subtracted the mean magnitudes across both rows and columns. Lastly, time-frequency bins with a magnitude below a chosen threshold (denoted NT) were set to 0; a range of thresholds was tested to select an optimal value. As the upcall is characterized by a narrow contour sweeping upwards in frequency, we anticipated for it to be robust to these subtraction steps. Finally, the phases of all time-frequency bins were set to the original values to preserve signal coherence, and the denoised spectrogram was mapped back to the time domain with an inverse Short-Time Fourier Transform. This denoising pipeline was applied to both the primary dataset of upcalls and the “control” dataset of ambient noise samples. Representative examples of upcall and noise samples are displayed in the leftmost columns of Fig 1A and 1B, and de-noised versions of these samples at different thresholds (NT) are shown in the following columns.

Then, to ensure that the classification results were representing characteristics of upcalls rather than of tag-specific noise, the analysis procedure was performed in parallel for the upcall samples and noise samples across a range of denoising thresholds. At the threshold for which the CV accuracy of the noise samples was near-random, we could conclude that upcall accuracy was truly indicative of individual identification.

### Frequency shifting

BirdNET embeddings were constructed as the model learned species-specific differences in bird calls and song. In consequence, these features could be expected to best represent the avian frequency range, and to likely have poorer sensitivity for acoustic features at low (<500 Hz) frequencies. To account for this effect, we applied and compared two techniques for increasing the frequency of the upcalls prior to calculating embeddings. First, we considered a simple compression of the signal, shortening the signal in time and proportionately scaling the frequency range. We considered scaling factors from 2 (yielding a duration of 1.5 sec and frequency range of 100–1000 Hz) to 20 (yielding a duration of 0.15 sec and frequency range of 1000–10000 Hz) in increments of 2. Additionally, we considered increasing the frequency by 1 kHz while keeping duration constant through complex modulation. Specifically, we calculated a new signal *y* with values given by:


$$\begin{array}{c} y[k]=\text{real}\left(e^{-2\pi i\frac{k\Delta f}{s}}\;x[k]\right) \end{array}$$

for all indices *k* = 1, ..., *N*, where Δf is the frequency shift to apply to the signal, *s* is the sampling rate, and *N* is the number of samples in the signal *x*. We again considered a range of shifts, from Δf=1 kHz to Δf=10 kHz in increments of 1 kHz. Lastly, a Butterworth high-pass filter with a cutoff of Δf was applied to *y*[*k*] to prevent aliasing. Visualizations of frequency scaling and shifting are shown in Fig 1C and 1D.

### Quantifying detection and recognition

After evaluating the ability to identify individuals across denoising parameters and frequency modifications, we aimed to quantify the effect of ambient noise levels on both detectability and individual recognition. First, we selected de-noised upcall samples for a denoising threshold of 0.4 and frequency modification of 12x, parameters at which upcall classification accuracy was highest while noise classification accuracy was near-random. Next, we added random background noise to achieve SNR values ranging from -30–30 dB at increments of 5 dB. Rather than generating random noise based on measured noise recordings, which contained tag-related artifacts, we represented typical ambient ocean noise with a pink noise spectrum. Specifically, we generated noise samples with magnitudes $A(f)=(f+100{)}^{-\alpha }$ where *f* denotes frequency in Hz, α was randomly sampled between 0.5 and 1.5, and phases were sampled uniformly at random in [0, 2π]. SNR was defined as the ratio of mean-square signal amplitude to noise amplitudes within the upcall band (50–500 or equivalent after frequency modification). For each SNR level, we repeated the above classification pipeline, calculating embeddings and quantifying CV scores. To quantify detectability, we evaluated binary classification, distinguishing upcall samples from noise samples, relative to a random accuracy of 50%. To quantify recognition, we evaluated individual classification, relative to a random accuracy of about 23% (the proportion of the largest class in the dataset). For robustness across random sampling, the generation of pink noise was repeated ten times, providing a distribution of cross-validation scores for each SNR level.

## Results

### Effects of denoising

The removal of ambient noise is demonstrated in Fig 1. In this example, this pre-processing approach effectively removed the overall ambient noise profile and persistent impulsive noise visible in the upcall recording. No harmonics above 500 Hz are visible due to a bandpass filter. By a threshold of 0.4, the noise sample becomes entirely collapsed to zero, while key aspects of the call sample are preserved.

### Effects of frequency shift

**Fig 2:**
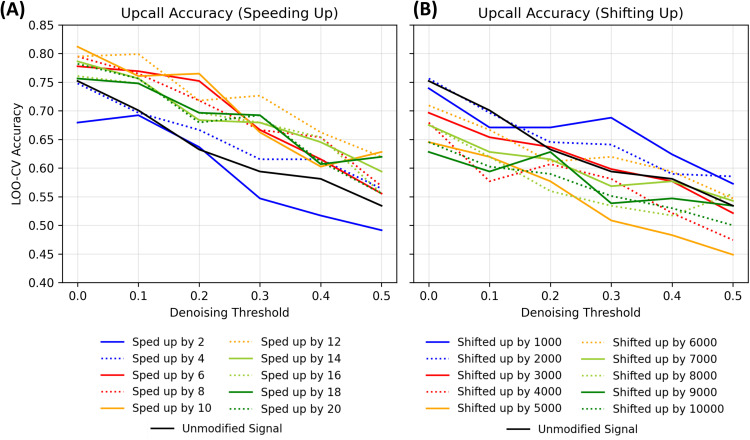
We quantified the effect of frequency modification on leave-one-out cross-validation (LOO-CV) upcall classification accuracy, comparing speeding up the signal (A) and shifting up the signal with complex modulation (B). Overall, speeding up yields higher performance for this dataset, likely because BirdNET is more sensitive to mid-frequencies vocalized by birds.

Visualizations of frequency modification for an example upcall, both by speeding up and shifting up, are displayed in Fig 1. Correspondingly, the leave-one-out CV accuracies across denoising thresholds for both forms of frequency modification are displayed in Fig 2. Overall, speeding up the calls improved classification, with an optimal speedup of 12x, while shifting up calls generally reduced performance for shifts above 2 kHz. We find that frequency scaling can give a modest but consistent improvement: accuracies for a scaling factor of 12x are about 10% higher than for the original sampling rate.

Curiously, these results agree closely with the anticipated sensitivity of a model trained for species-level bird song and call classification. At a speedup factor of 12x, the 50–500 Hz upcall is transformed to span the 600–6000 Hz range – broadly, the range most occupied by bird calls [94], particularly when considering high-frequency attenuation in PAM data. Similarly, a 1 kHz shift would move the upcall to span 1050–1500 Hz. However, simply shifting up the upcall would make it appear perceptually flatter, and likely more dissimilar to the calls used for training BirdNET. On the other hand, speeding up the call would proportionately expand the frequency range of the signal, potentially emphasizing subtle frequency characteristics. Moreover, a sped-up upcall becomes structurally similar to a brief chirp or alarm call, likely creating similarities to the signals on which BirdNET was trained. Overall, these results suggest robustness of BirdNET to new signals – particularly when appropriate augmentations are applied to help bridge domain shifts between training and deployment data.

### Classification performance across thresholds

**Fig 3:**
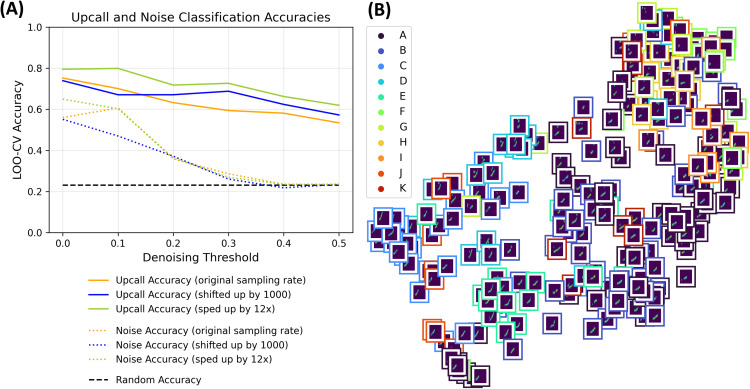
**(A)** Overall leave-one-out cross-validation (LOO-CV) accuracies for both the upcall dataset and the noise dataset at varying denoising thresholds. Accuracies for the upcall dataset are shown in solid lines, while accuracies for the corresponding noise dataset are shown in dotted lines. Additionally, color indicates the frequency modification applied to the audio, with the unmodified spectrum shown in orange, frequency modulation by 1 kHz shown in blue, and speeding up by a factor of 12 shown in green. **(B)** Two-dimensional UMAP projection of the upcall embeddings for a denoising threshold of 0.4 and a scaling factor of 12x. The color framing each spectrogram indicates the individual.

Fig 3A displays the leave-one-out cross-validation accuracies of the classification pipeline as a function of the denoising threshold. Solid lines indicate upcall accuracies, dashed lines indicate noise accuracies, and color indicates frequency modification. Overall, this figure suggests several key conclusions. Without any denoising, we obtain a classification accuracy of 84% for the upcall dataset and 79% for the corresponding noise dataset. When spectral subtraction is applied without thresholding (threshold = 0), the upcall and noise classification accuracies are 79% and 65%, respectively, for the optimal scaling factor of 12x. In other words, despite significant denoising, there were still sufficient tag-specific acoustic characteristics to differentiate the noise samples by tag. This finding underscores the difficulty of decoupling desired signals from confounding factors, a challenge exacerbated by the limited interpretability of deep learning methods. However, when spectral subtraction was followed by increasingly restrictive thresholding, the noise accuracy gradually decreased to the level of a random classifier, while the call accuracy remained high. Notably, at a threshold of 0.4, when the noise dataset is effectively random (24%), the call accuracy remains at around 66%, indicating that the upcall samples can be successfully differentiated by individual even after controlling for ambient noise.

To visualize the distribution of intra- and inter- individual variability across upcalls, Fig 3B presents a two-dimensional UMAP projection of the BirdNET embeddings for all calls at a threshold of 0.4. The colors of the spectrogram borders indicate individual identity. Overall, there is visible grouping of calls by individual whale, showing that individual variation is a major driver of variability in upcall structure. Some differences in upcall duration, slope, and frequency range are visually perceptible across the UMAP space.

### Effects of ambient noise

**Fig 4:**
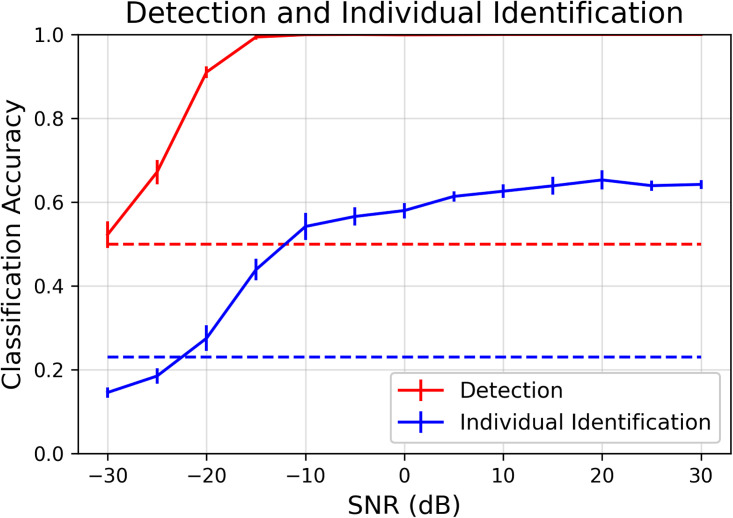
Classification accuracy of detection (in red) and individual identification (in blue) tasks across varying SNR for the upcall dataset. The solid lines indicate classification accuracies, and the dashed lines indicate random accuracy.

Fig 4 displays the accuracies for detection (in red) and recognition (in blue) as a function of SNR. At the lowest SNR of -30 dB, neither detection nor recognition is feasible. The detection curve increases sharply and reaches a plateau at about -15 dB. In contrast, the recognition curve remains near-random until -20 dB, followed by a more gradual increase, starting to plateau near -10 dB. The difference between these curves gives the difference in thresholds between detection and classification for the BirdNET model. To more directly compare these values, both curves can be normalized to span the range from 0 to 1 and linearly interpolated, such that this gap can be estimated at intermediate values of normalized accuracies. For instance, at the 50%, 75%, and 90% values of the maximum accuracy for detection and individual identification, the SNR thresholds for detection are -23 dB, -20.5 dB, and -17 dB (respectively) while the corresponding thresholds for identification are -16 dB, -10.5 dB, and 3.5 dB. Therefore, the threshold gaps at these values are 7 dB, 10 dB, and 20.5 dB.

## Discussion

### Detectability and communication space

A foundational study by Clark et al (2009) re-formulated the SNR threshold to represent different aspects of signal recognition, terming it the “recognition differential" (RD), defined by the addition of a detection threshold (DT) term (the SNR threshold of animal auditory perception) minus a signal processing gain (SG) (the improvement in detectability and/or recognition due to signal structure) and a directivity index (DI) term (representing binaural hearing gain) [24]. This formulation is flexible, and can be adapted for different forms of communication by appropriately adjusting the latter two terms. In their analysis, the authors set DT = 10 dB, DI = 0 dB, and SG = 16 dB, yielding an overall threshold of -6 dB that defined the outer boundary of the communication space. The significant differences in methodology between their study and our work make it challenging to directly compare threshold magnitudes. However, our work suggests that this threshold may not be sufficient for individual identification, which, for BirdNET, would require an additional ≥ 7 dB.

By assuming a propagation model for a given environment, the x-axis for the curves displayed in Fig 4 can be converted to units of distance (and could thereby yield an estimated *detection function* as considered in studies of distance sampling for density estimation [95]). The individual identification curve would similarly yield a recognition function – in essence, a probabilistic extension of a communication range. Considering an environment in which acoustic propagation can be approximated with simple spherical spreading (where intensity would decrease as $10\log_{10}(r^{2})$ with distance *r*), a 7 dB difference in intensity would correspond to a detection range $10^{7/20}\approx 2.2$ times greater than the individual identification range, and therefore a 2-dimensional detection space about $10^{7/10}\approx 5.0$ times greater than the individual ID space. In an shallow environment in which acoustic propagation can be instead approximated with simple cylindrical spreading (where intensity would decrease as $10\log_{10}(r)$ with distance *r*), this difference would imply a detection range and 2-dimensional area about 5 and 25 times greater than the respective range and area for individual identification. To further interpret Fig 4, suppose a noise source increases background noise levels and reduces the SNR of a call from -10 to -20 dB. Then, the detection accuracy would decrease from 100% to 91%, while the accuracy of individual identification would be approximately halved, from 54% to 28%. Therefore, considering detection alone creates a risk of underestimating anthropogenic impacts.

Given the critical state of the NARW population, particular attention has been dedicated to understanding the impacts of anthropogenic noise on their communication. Through a simulation grounded in real-world measurements, Clark et al (2009) found that the passage of a single commercial vessel can decrease the detection space by 97% [24]. Hatch et al (2012) analyzed passive acoustic recordings in the Stellwagen Bank National Marine Sanctuary to quantify acoustic masking due to both heightened ambient noise and shipping noise across a range of parameters [47]. Broadly, the authors find that relative to hypothesized historical ambient noise levels, the heightened underwater noise levels have led to 65% loss of the detection space during quiet periods, and 87% loss during noisy periods. By modeling acoustic propagation, Tennessen and Parks (2016) calculated that right whale upcalls will be entirely masked by noise if a container ship is within a 25 km range [96]. While right whales could compensate by increasing their call amplitude by 10 or 20 dB, this translates to a ten-fold or hundred-fold increase in amplitude: likely a significant energetic cost. Crucially, all of these studies ultimately evaluate the ability to detect calls, and not the capacity to perceive vital, more complex information such as individual identity. Computational estimation of communication parameters could begin to address these unknowns, and refine assessments of anthropogenic impact.

### Implications for acoustic individual identification

Our work contributes to, and closely aligns with, a broader context of acoustic individual identification (AIID) reviewed and conceptually formulated by Knight et al [97]. Of the 598 published studies of AIID reviewed in this work, 96% have indicated that individuals can be distinguished vocally, strongly suggesting that acoustic signatures can be found in most-sound producing taxa. However, while the review included 65 papers on “aquatic mammals,” almost all considered pinnipeds or odontecetes. Only three papers focused on individual identity in mystecetes, of which two examined humpback whales [98,99], accompanied by the NARW analysis of McCordic et al [79] which we continue in this work. Furthermore, of all studies considered in the review, less than 5% leveraged deep learning, likely due to the challenges of training models with low sample sizes. We hope that our work can contribute to the understanding of mysticete acoustic behavior, as well as provide a methodology for expanding the use of deep learning within AIID.

In the terminology of Knight et al, our analysis considers the “closed-set” problem of building a classifier to distinguish a known number of specific individuals, with “targeted” tag recordings. The associated “open-set” problem — determining the number of NARW present in an area from passive acoustic data — could enable greatly streamlined abundance estimation, and therefore would be highly applicable to conservation needs. However, this task is substantially more challenging: both in acquiring a ground-truth dataset to evaluate possible techniques, and in designing an abundance estimation methodology that would be accurate and robust to irrelevant dataset characteristics. For NARW, it could be feasible to construct a ground-truth open-set AIID dataset through appropriate augmentation of tag recordings or perhaps acoustic localization of passively-recorded upcalls, continuing prior work on abundance estimation [100]. Additionally, the denoising approach utilized here could similarly be applied to large-scale datasets to improve robustness to ambient noise conditions. However, in our analysis, the call samples had relatively high SNR, and were manually identified and cut, ensuring minimal overlap with other upcalls. For unsupervised use in a natural recording, an analysis pipeline would necessarily need to address co-occurring sound events. Moreover, a number of external factors may affect acoustic signatures, complicating the problem of individual identification, especially over broader spatial and temporal scales. In particular, NARW are known to refine upcall characteristics with age [79], particularly as calves and juvenile whales mature [101], and modify frequency and amplitude in response to ambient noise conditions [102]. Behavioral state, which in turn is closely tied to diel and seasonal cycles, is known to be associated with produced call types as well as vast variability in call rate [71]. Accordingly, it may likely also affect upcall characteristics. At different distances, effects of acoustic propagation will also attenuate and distort upcalls. Evaluating the influence of these factors is challenging, requiring a diverse and detailed dataset, and was not feasible in this case study. However, thorough understanding of such effects would be essential for robust AIID-based abundance estimation.

### Challenges and caveats

In this work, we quantified information transfer in the form of individual identification from a contact call. Of course, information is a highly context-dependent concept, and will vary by the caller, listener, signal characteristics, and form of information conveyed. Additionally, these results represent SNR in the presence of simulated pink noise; in practice, heterogeneous noise characteristics would otherwise affect these results. Lastly, SNR may have variable definitions, so quantitative values may not directly transfer across studies.

Deep learning opens new opportunities for bioacoustic analysis, but not without challenges and caveats. In this study, tag-specific ambient noise proved to be a significant confounding factor. When applying highly sensitive machine-learning-based tools, caution is warranted, particularly when ecological conclusions may be impacted by correlated patterns of ambient noise. Additionally, pre-trained models can effectively analyze small-scale datasets, enabling classification performance that would have been infeasible with prior machine learning methods. However, domain differences between the training and testing datasets may affect performance. In this study, we confirmed that a model trained on bird call and song recognition could be successfully applied to low-frequency whale calls, but found that scaling the calls to a bird-like frequency range improved classification accuracy.

## Conclusion

In this study, we demonstrate that deep-learning-based techniques can distinguish individual identity in a dataset of NARW upcalls – a call type considered to be heavily stereotyped. Moreover, by leveraging deep learning, we quantify the ability to recognize individual characteristics in a noisy environment, and thereby infer the loss of NARW communication space due to increased ocean noise levels. Overall, while our analysis focused on one species of critical conservation concern, the methods are general, and can be applied across taxa. Specifically, applying this workflow to other problems would require several choices: (1) identifying an ecologically-meaningful aspect of communication, (2) finding or constructing a ground-truth dataset, and (3) selecting a model for calculating audio embeddings. Fortunately, bioacoustic datasets on individual identity, call type, dialects, and other vocal characteristics are becoming more common and accessible, as are a collection of taxonomically-diverse high-performing models. Together, these developments open opportunities to communication spaces along these dimensions across taxa. In particular, we hope that this case study can serve as an example of a low-cost method for quantifying communication parameters for marine mammals, thereby improving estimates of acoustic masking and corresponding anthropogenic impact assessments.
