# Supplementary material for: Detection, communication, and individual identification with deep audio embeddings: A case study with North Atlantic right whales
Source: PLoS Comput Biol. 2026 Jun 30;22(6):e1013321. doi: 10.1371/journal.pcbi.1013321 (PMC13345461; doi:10.1371/journal.pcbi.1013321)
Supplement: Attachment — Submitted filename: review.pdf [file pcbi.1013321.s001.pdf]

In this study, the authors aim to use deep learning to better understand and quantify the gap between the “detection” space and the “recognition” or “discrimination” space within the broader “communication space.” These concepts are very interesting, and the idea that confusion between these spaces can lead to underestimating the impact of anthropogenic noise on marine mammals is a particularly compelling question. The context is well explained, and the description of past work helps ground the study within a well-documented history of deep learning applications in bioacoustics. However, this section could benefit from a stronger focus on marine mammals specifically. A clearer separation between the introduction and the “state of the art” sections might be a good idea, although their current integration does not significantly disrupt the reading.

The study focus on North Atlantic Right Whales for two reasons: they are an endangered species, making their conservation a priority, and they emit a particular call that conveys individual information, allowing researchers to “measure communication” (i.e., if the identity of the source is detected, there is no information loss and the communication is considered successful). The data used is retrieved from tags, and the dataset contains a relatively small number of samples, coming from only a few individuals. While this amount of data is sufficient to apply deep learning techniques, it raises the question of whether simpler signal processing methods, combined with statistical approaches (such as acoustic indices), might have been adequate. The authors seem to address this concern by stating that *“manually selecting acoustic metrics imposes assumptions and limits the patterns on the acoustic characteristics that can distinguish individuals”* which is true and can justify the use of deep learning—but the same argument also applies to data annotation. However, the annotation process is neither mentioned nor described, nor is the selection of the 3-second window. The paper would benefit significantly from including details about this part of the methodology, as well as general information about the dataset (such as class repartition and potential imbalance). Although the paper does not address this, it is also important to keep in mind that tags may record vocalizations from nearby whales; using the tag as a proxy for the sound source is common, but it can lead to misidentifying individuals.

To compensate for the limited dataset size, the authors chose to use transfer learning and encode the data with BirdNET. This choice is relevant, as BirdNET is a bioacoustic model; however, since primarily trained on bird vocalizations, a frequency gap issue arises. The authors successfully addressed this by scaling and shifting the low-frequency vocalizations of interest. The inputs are also preprocessed through denoising to prevent the network from learning tag-specific features. To further ensure this, a “control” set of noise is generated from the data, and performance is compared against it. This is a very good idea and is something that should be more commonly implemented in similar studies.

After pre-processing, vocalizations are processed by the BirdNET + SVM network. The choice of using an SVM “head” for classification could benefit from further justification. Ablation studies are conducted to compare the different pre-processing steps. Overall, the experiments appear thorough and well executed. Performance is assessed using accuracy, which is somewhat questionable, as relying on a single metric can be misleading. The use of PR and ROC curves is widespread in the community and would have allowed for more robust evaluation and easier comparison with related work. UMAP visualization is also used to illustrate individual clusters of calls, which is a good idea. However, drawing statistical conclusions from such methods should be done cautiously and would benefit from an analysis of the explained variance retained during dimensionality reduction.

The discussion is very interesting. Paragraph B, which links threshold results to physical measurements of the detection and recognition spaces, is particularly valuable, as it grounds the paper in its practical application and emphasizes its relevance. Paragraph C, which addresses challenges and caveats, is also insightful and shows that the authors have taken a critical step back from their work. Limitations and directions for future research are well identified.

However—and this is where the major issue lies—it is unfortunate that one of the paper's core assumptions is made explicitly clear only at this stage: *"Our analysis leverages the properties of audio embeddings as a proxy for the perceptive capabilities of NARW."* This assumption is central to the work and quite ambitious. It is justified by the fact that neural networks have demonstrated strong performance, sometimes even outperforming humans in audio discrimination tasks—but this does not automatically extend to marine mammals. Furthermore, the sounds fed to the network were scaled and/or shifted, meaning they were not "heard" in the way they are actually emitted and perceived in the ocean. Deriving thresholds from neural network performance may indeed be an interesting first step—especially given the urgent conservation needs of nearly all whale species—but treating network performance as a reliable indicator of perceptual thresholds is a significant leap and must be strongly justified.

Overall, the paper is well-written and easy to follow. Some acronyms would benefit clearer explanation (e.g. NT in caption of the figure 1, or LOO in the caption of figure 2 and 3).
