# Supplementary material for: Detection, communication, and individual identification with deep audio embeddings: A case study with North Atlantic right whales
Source: PLoS Comput Biol. 2026 Jun 30;22(6):e1013321. doi: 10.1371/journal.pcbi.1013321 (PMC13345461; doi:10.1371/journal.pcbi.1013321)
Supplement: Attachment — Submitted filename: NARW_ID__Response.pdf [file pcbi.1013321.s002.pdf]

Authors' Response to Reviews of

Detection, communication, and individual identification with deep audio embeddings: A case study with North Atlantic right whales

Irina Tolkova, Holger Klinck, Dana A. Cusano, Anke Kügler, Susan E. Parks

PLOS Computational Biology, PCOMPBIOL-D-25-01388

RC: *Reviewers' Comment*, AR: Authors' Response

We sincerely thank the reviewers and editorial team for their thoughtful feedback. We have revised the manuscript according to their recommendations, and hope these changes have addressed all reviewer concerns. Below, we have included a copy of each reviewer's comments (indicated as RC) and provided point-by-point responses to each (indicated as AR). The relevant line numbers for each revision are indicated in blue and refer to the tracked-changes document. We have also reformatted the revised manuscript for the PLOS template.

Additionally, we would like to make note of several additional changes to the manuscript. First, all of the individual NARW have been anonymized in the associated data and figures for this work, to protect the critically endangered species from possible misuse of their identifying information. The dataset and associated code is now publicly available at this GitHub repository: https://github.com/avokloti/narw-acoustic-identification. In finalizing the code as part of this review, we made a minor change (bandpass-filtering audio samples prior to normalizing, rather than after normalizing, which is conceptually more meaningful) which influenced the relative magnitudes of the denoising threshold and the quantitative values in Figure 3, but did not impact any of the overarching conclusions. We believe these modifications, alongside the revisions described below, have improved the quality of the manuscript, and hope they have addressed all reviewer questions and comments.

Editor

RC: *The reviewers are in agreement that this paper represents an interesting contribution to the literature, empirically demonstrating a useful point about animal communication in noise. There are many reviewer comments, and all should be addressed in the resubmission. The reviewers are very much in agreement with each other, so I do not need to provide any further guidance - I trust you will find the reviews helpful.*

AR: Thank you to the reviewers and editorial team for their consideration of this manuscript.

Reviewer #1

Comment 1.1

RC: *This work presents a novel perspective on using deep audio embeddings for conservation purposes, by proposing a framework to estimate the communication space of a species, going beyond detection. This shift in paradigm is an important and necessary contribution to the field. Also, the efforts made to eliminate the background noise as a confounding factor is commendable, and rare enough to be highly appreciated. What mainly lacks still is the completion of the introduction regarding:*

- *why would the individual identity information be of importance to the NARW*
- *prior works of modelling intelligibility with deep audio embeddings*
- *other existing bioacoustic deep audio embedders*

AR: Thank you very much for your time and suggestions in reviewing this manuscript. Our revisions for each of the three identified recommendations are described in the associated comments below.

Comment 1.2

RC: *"Models trained on bioacoustic data..." Since models is plural here, could you mention the other networks that have been proposed for such purposes? I am thinking of Hagiwara 2023 and Best 2022 for instance. Regrettably, I have not seen a comparison of Birdnet and these two models (there might be more), despite they all claim to serve the same purpose of generic deep embddings for bioacoustic data. Comparing them for this use case would be great, but you might deem it to be out of scope for this study. Since deep audio embeddings are central to this paper, I believe an introduction on the varying models that exist and how they differ (e.g. training procedure, training data) is necessary. "AVES: Animal Vocalization Encoder Based on Self-Supervision" Hagiwara 2023 "Deep audio embeddings for vocalization clustering" Best et al. 2022*

AR: Thank you for this suggestion, we added an additional section to describe and contextualize different models for calculating embeddings. As there are now a wide variety of bioacoustic foundation models, and a reader can find excellent discussions and evaluations of different models in other papers (such as Schwinger et al 2025: https://arxiv.org/pdf/2508.01277), we do not aim for this discussion to be comprehensive, but rather to point the reader to useful resources.

Revisions in lines: 282-309

Comment 1.3

RC: *"In this light, machine learning could also provide a new opportunity to quantify information transfer within animal vocalizations, filling a key gap in the study of communication." The connection between previous findings on the capability of deep embeddings to discriminate between calls, species or individuals, and the fact that they can model perception abilities in non-humans seems too weak. After a quick search, it seems this question has been already studied for birds (Morfi et al. 2021) and for humans (Haro et al. 2020, Best et al. 2024). Specifically for the research question of this paper, the field of speech intelligibility prediction seems appropriate to explore/refer to. The previous argumentation gives a great introduction, which just needs to be completed with a review of literature on modelling sound perception with deep learning models. "Deep perceptual embeddings for unlabelled animal sound events" Morfi et al. 2021 "Deep Neural Network Model of Hearing-Impaired Speech-in-Noise Perception" Haro et al. 2020 "Transfer learning from Whisper for microscopic intelligibility prediction," Best et al. 2024*

AR: Thank you very much for these references! We followed your recommendation to expand our discussion with a overview of how deep learning models have been used for modeling sound perception.

Revisions in lines: 700-748

Comment 1.4

RC: *"As all whales, NARW rely on sound for communication" perhaps 'repertoire' is more appropriate here than 'vocabulary'? Besides the mother-calf bond maintenance mentioned later, are there other functions to this communication that make it vital for the whales?*

AR: Thank you for this point. We made the suggested revision, and elaborated on the significance of individual identification for both mother-calf pairs and for broader social bonds.

Revisions in lines: 211-233

Comment 1.5

RC: *"The impacts of acoustic masking have wide-ranging impacts on a social species" Maybe remove "The impacts of"?*

AR: Thank you for catching this. We rephrased this section.

Revisions in lines: 197-203

Comment 1.6

RC: *"Recordings of upcalls therefore provide..." Could you discuss why/how individual identity could be important to the whales? It seems crucial for the claims of this paper. If a higher SNR is necessary for individual ID as compared to call type discrimination, and if only the call type information actually matters to the whales, the communication space would be underestimated. Depending on the hypotheses on the importance of individual ID, could you make reference to the framework of Dooling et al. to classify in which category do you place the space in which individual ID is possible?*

AR: Thank you for this comment, we elaborated on the significance of individual identification for NARW.

Revisions in lines: 211-233

Comment 1.7

RC: *"quantify communication" This seems odd, could you reformulate?*

AR: Thank you, we have rephrased this sentence.

Revisions in lines: 235

Comment 1.8

RC: *"adapting to lower-frequency data" adapting what?*

AR: We were referring to modifying the frequency range, as discussed later in the manuscript; we have modified this sentence for clarity.

Revisions in lines: 253-254

Comment 1.9

RC: *"...within prior estimates, ..." Maybe replace estimates with findings?*

AR: Modified, thank you.

Revisions in lines: 255

Comment 1.10

RC: *"standardized to a length of 3 seconds by zeropadding." Why is this necessary? Would it not be possible to simply extract 3 seconds of signal around each call? This seems particularly important if the duration of the call carries individual information as stated later. If calls are pre-cut manually and zero-padded, it would be a strong help to the following pipeline and has to be mentioned.*

AR: In this analysis, we are aiming to evaluate the degree to which individual identity is encoded in NARW upcalls. We therefore need to ensure that our results are reflective of individual characteristics; our analysis would be misrepresentative if the model was learning features of other sound events within a 3-second window. We definitely agree with the reviewer that this is a "strong help" to the analysis; a system aiming to perform automated individual identification within a natural soundscape would have to address these challenges. We emphasized this point in the discussion of AIID.

Revisions in lines: 892-897

Comment 1.11

RC: *Could you specify the median and std for number of calls per individual?*

AR: Yes, thank you – the number of calls per individual varied from 10 to 54 with a median of 12, and we added this in the text.

Revisions in lines: 326 - 327

Comment 1.12

RC: *If I understand correctly, all calls from a given individual were collected with one tag. If deemed reasonable, please discuss the possibility that the variation between upcalls could arise from behavioral variations instead of individual signatures (e.g. if one individual was more stressed than another during the tag recording, this could generate upsweep shape differences in this dataset which would not appear in recordings from another context).*

AR: Behavioral variations are certainly a factor which could cause variation in individual upcall characteristics, alongside age, sex, and other factors. However, it is difficult to obtain behavioral observations alongside focal upcalls, making this challenging to study. We added an additional emphasis on behavioral variation in the discussion of AIID.

Revisions in lines: 904-908

Comment 1.13

RC: *"In fact, BirdNET embeddings have yielded higher classification accuracies for downstream bioacoustic tasks..." Again here, it would seem fair to mention other models proposed for embedding bioacoustic data.*

AR: Thank you, we have added a brief overview of the space of embedding models to the text.

Revisions in lines: 282-309

Comment 1.14

RC: *"BirdNET Analyzer v1.5.1" Could there be a citation to ease finding this software?*

AR: Definitely, we added an additional reference to the main BirdNET publication, the main citation associated with this software.

Revisions in lines: 396

Comment 1.15

RC: *"vectors of 1024 features for each audio sample" Does this imply a mean pooling of the 3 seconds excerpts? This is crucial and should be detailed and discussed.*

AR: Yes; the audio embeddings describe the full 3-second segment, resulting in one embedding vector per upcall. This form of pooling has been effective in other bioacoustic analysis tasks. We added additional clarification of this point in the text.

Revisions in lines: 397-398

Comment 1.16

RC: *"with these acoustic features" It seems odd to describe audio embeddings as acoustic features, since the term is often used to describe formalised acoustic measurements.*

AR: Here, we meant the term "features" broadly, as it seems used in the literature – as any numerical representation of data samples, whether from manually-derived measurements or from learned representations. We added a brief clarification in the text.

Revisions in lines: 275-278, 394

Comment 1.17

RC: *"we could infer that tag-specific audio characteristics were a significant driver of the variation in upcall samples." This sentence seems more linked to section III C, I don't understand the connection to the rest of the paragraph.*

AR: The intent of the sentence was to motivate the purpose of these methods; but we agree with the reviewer and removed it from this paragraph. Thank you for the suggestion.

Revisions in lines: 405 - 408

Comment 1.18

RC: *"BirdNET embeddings were learning" It seems that learning is not accurately used here.*

AR: Thank you, we rephrased this sentence.

Revisions in lines: 427

Comment 1.19

RC: *"classification pipeline this additional" Missing 'to'?*

AR: Thank you, corrected.

Revisions in lines: 434

Comment 1.20

RC: *Could Figure 1 be mentioned already in the methods section to help readers understand the procedures described?*

AR: Thank you for the suggestion. We added references to Figure 1 when describing denoising and frequency modifications to aid the reader in visualizing these processing steps.

Revisions in lines: 465-467, 501-502

Comment 1.21

RC: *In Fig.3 B, I understand that showing upcall spectrograms can help interpret the figure but it also makes it difficult to see clusters with overlapping snipets. I believe individual positions in the low dimensional space here is more important than visualising patterns in the snipets which is hardly doable. Could you consider switching to points, maybe with some transparency to avoid masking in case of overlaps?*

AR: We do feel that the spectrograms are a key aspect of this plot, as the points alone are not particularly interesting – as clear from the leave-one-out accuracies, we do not expect separable clusters in this analysis, particularly not in an unsupervised 2D space. We have tried to scale and modify the visualization to make the spectrograms, and patterns of individual variability, easier to see.

Revisions in lines: Figure 3B

Comment 1.22

RC: *"we selected de-noised upcall samples for the best performing denoising threshold" Isn't the best performing denoising threshold also a threshold at which classification is possible from background noise alone?*

AR: We intended to describe the parameters at which upcall classification accuracy was highest while noise classification accuracy was near-random, and clarified this in the text. Thank you for pointing out the ambiguous language.

Revisions in lines: 508-512

Comment 1.23

RC: *Section III E Using more realistic noise instead of white noise (boat sound or background noise from the DTAGS for instance) would improve the strength of the analysis here.*

AR: Thank you for this suggestion for improving our analysis. We first considered augmenting call samples with randomly-sampled time series from the distribution of DTAG background noise. However, this distribution had distinctive peaks at that were clearly associated with the tags, and not typical of marine background noise profiles. As the goal of our analysis was to represent acoustic masking by ambient underwater noise, we revised the analysis to instead consider pink noise within a range of randomly-sampled parameters, and accordingly revised the section on SNR analysis.

Revisions in lines: 512-524

Comment 1.24

RC: *"these results suggest robustness of BirdNET to new signals" The results discussed here show the impact domain knowledge can have in adapting signals for a given model, rather than the direct robustness of BirdNet.*

AR: Domain knowledge was certainly central to this analysis – thank you for this point. At the same time, BirdNET was able to distinguish individual NARW even without frequency modification, which is remarkable for a model that was trained solely for species-level bird song and call classification. We slightly elaborated on this sentence.

Revisions in lines: 583-585

Comment 1.25

RC: *"Accuracies for the upcall dataset are shown in solid lines, ..." IMO this belongs more to the legend of the figure.*

AR: We agree; we moved these sentences to the figure's legend, leaving a brief reminder in this paragraph.

Revisions in lines: Figure 3A caption, 592-599

Comment 1.26

RC: *Could you add the information of performance for the upcall and the noise datasets in the case of no spectral substraction? Ideally in Figure 3 left, or at least in the text?*

AR: Certainly – we have added these values in the text.

Revisions in lines: 600-602

Comment 1.27

RC: *"correlated factors" Would confounding factors be more appropriate here?*

AR: Thank you, modified.

Revisions in lines: 611

Comment 1.28

RC: *Are the embeddings in Figure 3 B from the original sampling rate or the sped up (most accurate) version?*

AR: The latter; we added a clarification in the caption. Thank you for pointing out this missing detail.

Revisions in lines: Figure 3B caption.

Comment 1.29

RC: *"the smaller separate cluster at the edge of the figure" It is unclear to me to what this corresponds to.*

AR: We removed this description from the text, as it became irrelevant after some revisions.

Revisions in lines: 631 - 634

Comment 1.30

RC: *"However, this task is substantially more challenging..." I do not agree with this statement. Although I agree applying abundance estimate to tag recordings is conceptually absurd, the dataset used in this study could be used to evaluate audio embeddings in an open-set setting, simply by switching from training an SVM and measuring accuracy to applying a clustering algorithm and measuring the NMI or the number of missed / double counted individuals. Augmenting the data with SNR variations, we could evaluate audio embeddings for an open-set AIID task and inform on their applicability to PAM recordings.*

AR: Yes, we agree with these points; the intent of this statement was that the closed-set problem is simpler than the open-set problem (where the number of individuals is unknown). To clarify this, we added the reviewer's suggestion about tag data augmentation to the text.

Revisions in lines: 885 - 888

Comment 1.31

RC: *"However, a number of external factors may affect acoustic signatures," In reference to the previous comment regarding this, can you discuss these factors not only for the potential collection of PAM AIID data, but also for the dataset used here?*

AR: Thank you for this comment. It was not feasible to evaluate the statistical influence of these factors in our dataset due to its small size and limited spatiotemporal scope. For instance, the suction-cup on-animal tags can only record for a few hours, and therefore are not suited to evaluate change in individual vocal characteristics over time, or the effects of diel cycle or seasonality. Our goal here is to emphasize that, while difficult, these factors would be critical to quantify before drawing conclusions from AIID-based abundance estimation. We clarify this in the text.

Revisions in lines: 910-912

Comment 1.32

RC: *The first paragraph of Section V B is difficult to understand and hard to put in context.*

AR: We have rephrased both the results and discussion relating to this paragraph for greater contextualization and clarity.

Revisions in lines: 653-670, 796-824

Comment 1.33

RC: *Could you add a quick summary to the main findings regarding the gap between detection and recognition and the corresponding "communication space" reduction in the conclusion and abstract?*

AR: We have added an additional sentence on the detection-recognition gap to the abstract, and elaborated on the gap significantly in the discussion for clearer interpretation of these results.

Revisions in lines: 29-30, 796-824

Reviewer #2

Comment 2.1

RC: *In this study, the authors aim to use deep learning to better understand and quantify the gap between the "detection" space and the "recognition" or "discrimination" space within the broader "communication space." These concepts are very interesting, and the idea that confusion between these spaces can lead to underestimating the impact of anthropogenic noise on marine mammals is a particularly compelling question. The context is well explained, and the description of past work helps ground the study within a well-documented history of deep learning applications in bioacoustics. However, this section could benefit from a stronger focus on marine mammals specifically. A clearer separation between the introduction and the "state of the art" sections might be a good idea, although their current integration does not significantly disrupt the reading.*

AR: Thank you very much for your time on this manuscript! In agreement with comments from another reviewer, we separated the section on state-of-the-art deep learning methods, now located after the introduction. Since the key conceptual methodology is relevant for both marine and terrestrial communication, we do not limit the scope of the paper to marine mammals, but emphasized that marine animals are particularly at risk from adverse impacts of anthropogenic noise.

Revisions in lines: Section 2, 48-54

Comment 2.2

RC: *The study focus on North Atlantic Right Whales for two reasons: they are an endangered species, making their conservation a priority, and they emit a particular call that conveys individual information, allowing researchers to "measure communication" (i.e., if the identity of the source is detected, there is no information loss and the communication is considered successful). The data used is retrieved from tags, and the dataset contains a relatively small number of samples, coming from only a few individuals. While this amount of data is sufficient to apply deep learning techniques, it raises the question of whether simpler signal processing methods, combined with statistical approaches (such as acoustic indices), might have been adequate. The authors seem to address this concern by stating that "manually selecting acoustic metrics imposes assumptions and limits the patterns on the acoustic characteristics that can distinguish individuals" which is true and can justify the use of deep learning—but the same argument also applies to data annotation. However, the annotation process is neither mentioned nor described, nor is the selection of the 3-second window. The paper would benefit significantly from including details about this part of the methodology, as well as general information about the dataset (such as class repartition and potential imbalance). Although the paper does not address this, it is also important to keep in mind that tags may record vocalizations from nearby whales; using the tag as a proxy for the sound source is common, but it can lead to misidentifying individuals.*

AR: Thank you for bringing up these points.

In this work, we used a subset of the dataset thoroughly described in McCordic et al (2016), so we avoided repeating the description of the annotation process, and added brief clarification in the text. We hope readers will consult McCordic et al (2016) for additional detail (lines 316-318). The dataset is indeed imbalanced, and we also added the range and median in the text. Additionally, expert annotators reviewed the upcall examples to ensure that they only included high-SNR upcalls produced when tagged whales were alone during focal follows, giving high confidence that they were associated with the tagged individuals.

Revisions in lines: 317, 325-327

Comment 2.3

RC: *To compensate for the limited dataset size, the authors chose to use transfer learning and encode the data with BirdNET. This choice is relevant, as BirdNET is a bioacoustic model; however, since primarily trained on bird vocalizations, a frequency gap issue arises. The authors successfully addressed this by scaling and shifting the low-frequency vocalizations of interest. The inputs are also preprocessed through denoising to prevent the network from learning*

tag-specific features. To further ensure this, a "control" set of noise is generated from the data, and performance is compared against it. This is a very good idea and is something that should be more commonly implemented in similar studies.

AR: Thank you for the thoughtful read of this manuscript.

Comment 2.4

RC: *After pre-processing, vocalizations are processed by the BirdNET + SVM network. The choice of using an SVM "head" for classification could benefit from further justification. Ablation studies are conducted to compare the different pre-processing steps. Overall, the experiments appear thorough and well executed. Performance is assessed using accuracy, which is somewhat questionable, as relying on a single metric can be misleading. The use of PR and ROC curves is widespread in the community and would have allowed for more robust evaluation and easier comparison with related work. UMAP visualization is also used to illustrate individual clusters of calls, which is a good idea. However, drawing statistical conclusions from such methods should be done cautiously and would benefit from an analysis of the explained variance retained during dimensionality reduction.*

AR: We agree with the reviewer that, generally, considering multiple metrics gives a more informed picture of a system's performance. In this case, the primary goal of our evaluation was not to present a performance comparison to other models, but rather to consider the effects of different parameters on individual identification. To this end, we chose to show accuracy, as it is a interpretable summary metric with a clear baseline for random performance. Additionally, PR and ROC curves are defined for binary classification problems; an alternative may have been a multiclass area-under-the-curve (AUC) value, averaged in a one-vs-rest approach, but we felt this would come at a disadvantageous cost of interpretability.

We agree with the reviewer's point about drawing statistical conclusions from dimensionally-reduced UMAP values, and do not do so in this work; our use of UMAP is solely for visualizing the underlying structure of the dataset.

Comment 2.5

RC: *The discussion is very interesting. Paragraph B, which links threshold results to physical measurements of the detection and recognition spaces, is particularly valuable, as it grounds the paper in its practical application and emphasizes its relevance. Paragraph C, which addresses challenges and caveats, is also insightful and shows that the authors have taken a critical step back from their work. Limitations and directions for future research are well identified.*

AR: Thank you!

Comment 2.6

RC: *However—and this is where the major issue lies—it is unfortunate that one of the paper's core assumptions is made explicitly clear only at this stage: "Our analysis leverages the properties of audio embeddings as a proxy for the perceptive capabilities of NARW." This assumption is central to the work and quite ambitious. It is justified by the fact that neural networks have demonstrated strong performance, sometimes even outperforming humans in audio discrimination tasks—but this does not automatically extend to marine mammals. Furthermore, the sounds fed to the network were scaled and/or shifted, meaning they were not "heard" in the way they are actually emitted and perceived in the ocean. Deriving thresholds from neural network performance may indeed be an interesting first step—especially given the urgent conservation needs of nearly all whale species— but treating network performance as a reliable indicator of perceptual thresholds is a significant leap and must be strongly justified.*

AR: The other reviewers have also emphasized the need for additional discussion and justification for the implication of embeddings for NARW perception. We have substantially expanded on the discussion section focusing on perception, and moved it to the start of the discussion.

Revisions in lines: 672-748

Comment 2.7

RC: *Overall, the paper is well-written and easy to follow. Some acronyms would benefit clearer explanation (e.g. NT in caption of the figure 1, or LOO in the caption of figure 2 and 3).*

AR: Thank you; we added clarifications of both acronyms in the figure captions.

Revisions in lines: Figure 1, 2, 3 captions

Reviewer #3

Comment 3.1

RC: *This work focuses on how to measure the impact that human-made noise has on the communication ability of vocal animals. Importantly, the authors describe assessing communication ability as more complex than simply detecting signals, and use the problem of individual identification in North Atlantic Right Whales (NARW) to exemplify this crucial distinction. In short, deep learning models are used to encode the calls, and identification accuracy is measured under several levels of added noise. The authors show that basing impact measures purely on detection performance leads to a significant underestimation, and that deep learning models can capture the complex particularities of the signals needed for effective information exchange.*

AR: Thank you for your time and recommendations on this manuscript.

Comment 3.2

RC: *Overall, the manuscript is clearly written and describes a thoroughly considered project. I find it detracts somewhat that the authors only address the clear difference between NARW ID recognition ability and audio embeddings at the end of the manuscript. While they mention that using audio embeddings as "a proxy for the perceptive capabilities of NARW" is an "imperfect comparison," the justification could be improved and advantages highlighted. Finally, a reflection on how this assessment could be applied to other forms of communication and species would help move beyond the specific case study and describe a more generalised process for evaluating the impact of human-made noise.*

AR: Thank you! The other reviewers have also emphasized the need for additional discussion and justification for the implication of embeddings for NARW perception. We have expanded on the discussion section focusing on perception, and moved it to the start of the discussion. Lastly, we have also emphasized that the steps for this methodology are general: provided examples of individually-identifiable calls and noise samples, this framework can be used with any embedding model of choice.

Revisions in lines: 672-748, 958-969

Comment 3.3

RC: *[Abstract] "communication space -the area.." –> instead of area, should it be space?*

AR: Our intent with this choice of words was to emphasize that "space" is a real, physical entity (not a figurative one); we kept "area" in the abstract but used the "space" phrasing in the main text, where there was opportunity to elaborate. Thank you for pointing this out.

Comment 3.4

RC: *[INTRO p.3] "we aim to quantify communication.." –> unclear or too vague.*

AR: Thank you, we rephrased this sentence.

Revisions in lines: 239-242

Comment 3.5

RC: *[III-A] " vectors of 1024 features for each audio sample" –> not clear what is an audio sample here, 3 seconds of audio? is the final embedding averaged over time?*

AR: Yes, audio sample refers to a short segment of a call or corresponding noise segment, standardized to 3 seconds. We clarified this point.

Revisions in lines: 397 - 398

Comment 3.6

RC: *[III-B] "low sample size..." –> dataset size?*

AR: We added this correction.

Revisions in lines: 415

Comment 3.7

RC: *[IV-D] "intermediate values..", not clear what values means here, accuracy values?*

AR: Yes, we intended to mean intermediate values of the normalized accuracies, and clarified this point in the text.

Revisions in lines: 663-664
