# Supplementary material for: Detection, communication, and individual identification with deep audio embeddings: A case study with North Atlantic right whales
Source: PLoS Comput Biol. 2026 Jun 30;22(6):e1013321. doi: 10.1371/journal.pcbi.1013321 (PMC13345461; doi:10.1371/journal.pcbi.1013321)
Supplement: Attachment — Submitted filename: review2.pdf [file pcbi.1013321.s003.pdf]

Introduction section : no major changes, overall looks OK for me. I just feel that paragraphs between page 1 and 2 could benefit from a better organization and structure, like we zoom in on NARW (« Specifically, we present... ») to then zoom out (« Vocally distinguishing... ») and then in again. Good to have include visual surveys and example observations. I appreciated the inclusion of visual surveys observations.

Background section : thanks for seperating the state-of-the-art section from the introduction. I feel like some references are still missing (like « deep learning revolutionized bioacoustics » is a big statement, even if I agree with it, a reference here would be nice, eg Stowell et al., 2022).
I think the frasing « embedding models » (l.282)is not the best one, I would say « embedding generators » or something like this, but it's a bit picky.
The list of the end of page 3 is a bit rough, I would appreciate a bit more « in deep » presentations, as well as some mentions of results obtained. I would also put the reference to Schinwger at the end, like « for more detailed work, see... »
I appreciated that the choice of BirdNet was further motivated.

Data acquisition section : thanks for including the fact that the annotation process is described in the original article, even though I think that's it could be a bit deepen in the paper, as it presents deep learning models. The annotation are the ground truth and the input of the model(s), it should be very clear what they are.

Method section : I would shorten the first sentence « Our methodology... » to just state « Our methodology entains four analytical steps : first…. » because here, some things are well motivated in the intro / background (the computation of embeddings to evaluate BirdNet's transfer learning abilities, the evaluation of individual indentifiability to evaluate how « ecologically-meaningful » is the communication), but some seems to be a bit less obvious (why modifying frequencies ? Why studying SNR ?), and then well motivated in the next paragraphs.

Section A : good to have specified how embeddings were computed. I think it would be beneficial to motivate the SVM-head choice (why not choosing a MLP for instance?)

Section B : l434 « above classification pipeline » l. 434 : which one ? The embeddings + SVM one ? It just lacks a bit of clarity here, as many pieces of interesting info are given. I really like how the noise was taken into account and how you spent time making sure it was not biasing the performances.

Section C : no major changes, ok for me.
Section D : no major changes, ok for me.

Results section :
Section A : no major changes, ok for me.

Section B : l. 570 « broadly the range most occupied by bird calls », this statement would benefit from a reference I think.

Section C : no major changes, OK for me.

Section D : I really like the fact that detection and classification tasks were led separately, to underlines what whas said in the intro about the different perceptual spaces. Interesting to see the curves not converging at the same moment. I agree that « the difference between these curves gives the difference between detection and classification », but I would mention that this is true for the

model, and not automatically for the animals (and maybe mention that this point is discussed in the discussion section right after)

Discussion section :

Section A : adding this section was definitely essential for the study, thank you for doing it. l. 694 « in the light of the strong parallel » would benefit from references. I am still a bit skeptical about the use of DNN as proxies, and the fact that DNN have proven to be good proxies for human auditory perception does not mean it's gonna work for marine mammal IMO. We do not have the same perceptual space (Uexküll's notion of umwelt). However, the mention of this is interesting, as by automatically detect and classify signal, one could say they are trying to mimick the work of human annotators. I think I would rather draw the parallel here than saying that because DNN suceed in mimicking human auditory perceptions, then it is relevant for marine mammals. This is especially true since signals were transformed (frequency shifted, denoised, etc., which is interesting and well motivated but which pulls them away from « natural » sounds).

Section B : I really like the parallel draw between the results presented and the ones that could be found in desnity estimation studies, through physics and propagation models.

Section C : I really like the closed-set to open-set perspective

Section D : No major changes, ok for me
