# Supplementary material for: Detection, communication, and individual identification with deep audio embeddings: A case study with North Atlantic right whales
Source: PLoS Comput Biol. 2026 Jun 30;22(6):e1013321. doi: 10.1371/journal.pcbi.1013321 (PMC13345461; doi:10.1371/journal.pcbi.1013321)
Supplement: Attachment — Submitted filename: NARW_ID__Response_to_Reviewers__Revision2.pdf [file pcbi.1013321.s004.pdf]

Authors' Response to Reviews of

Detection, communication, and individual identification with deep audio embeddings: A case study with North Atlantic right whales

Irina Tolkova, Holger Klinck, Dana A. Cusano, Anke Kügler, Susan E. Parks

PLOS Computational Biology, PCOMPBIOL-D-25-01388

RC: *Reviewers' Comment*, AR: Authors' Response

We sincerely thank the reviewers and editorial team for their thoughtful feedback. We have revised the manuscript according to their recommendations, and hope these changes have addressed all reviewer concerns. Below, we have included a copy of each reviewer's comments (indicated as RC) and provided point-by-point responses to each (indicated as AR). The relevant line numbers for each revision are indicated in blue and **refer to the tracked-changes document for clarity**. Additionally, a full list of revisions appears at the end of the tracked-changes document, after the references.

Editor

RC: *Thank you for your work on the resubmission. The 3 reviewers are in broad agreement that the improvements that have been made are good. However, all 3 reviewers also raised minor issues that should still be addressed before proceeding. Please do attend to these and resubmit.*

AR: Thank you very much to the reviewers and editorial team for their consideration and feedback on this manuscript.

Reviewer #1

Comment 1.1

RC: *comments 1.15 and 3.5: Two reviewers have asked for clarification on the pooling of embeddings within 3 second excerpts, but the information was still not added. It would be great if authors could give more details on the procedure of embeddings generation, without asking readers to go through the command-line interface documentation.*

AR: Sorry for misunderstanding this comment. The input to the BirdNET model is already a three-second audio signal, which agrees with the durations of the NARW call samples, so we do not implement any additional pooling. For greater clarity, we added clarification of BirdNET's pipeline and architecture, as well as further detail on the dataset.

Revisions in lines: 178-188, 339-341

Comment 1.2

RC: *- comment 1.22: Thanks for clarifying, could you still add the specific denoising threshold used?*

AR: Certainly, added. Apology for missing this in the first review.

Revisions in lines: 412

Comment 1.2

RC: *- The reference is missing for the first two mentions of clark et al (2009)*

AR: Thank you for catching this, added both references.

Revisions in lines: 208, 548

Comment 1.2

RC: *- "25 times greater then" should this be "greater than"?*

AR: Thank you, corrected.

Revisions in lines: 576

Comment 1.2

RC: *- "an 7 dB difference" should this be "a 7 dB difference"?*

AR: Thank you again, corrected.

Revisions in lines: 569

Reviewer #2

Comment 2.1: Introduction

RC: *Introduction section : no major changes, overall looks OK for me. I just feel that paragraphs between page 1 and 2 could benefit from a better organization and structure, like we zoom in on NARW (« Specifically, we present... ») to then zoom out (« Vocally distinguishing... ») and then in again. Good to have include visual surveys and example observations. I appreciated the inclusion of visual surveys observations.*

AR: To address this comment and Comment 3.1, we expanded the Background section into multiple subsections, and streamlined the Introduction. We hope this improves flow and readability.

Revisions in lines: Introduction and Background sections.

Comment 2.2: Background

RC: *Background section : thanks for seperating the state-of-the-art section from the introduction. I feel like some references are still missing (like « deep learning revolutionized bioacoustics » is a big statement, even if I agree with it, a reference here would be nice, eg Stowell et al., 2022). I think the frasing « embedding models » (l.282)is not the best one, I would say « embedding generators » or something like this, but it's a bit picky.*
The list of the end of page 3 is a bit rough, I would appreciate a bit more « in deep » presentations, as well as some mentions of results obtained. I would also put the reference to Schinwger at the end, like « for more detailed work, see... »
I appreciated that the choice of BirdNet was further motivated.

AR: Certainly. We added the suggested reference, and developed the background on embeddings, with both overviews of differences between models and results from comparative studies.

Revisions in lines: 124-202

Comment 2.3: Data acquisition

RC: *Data acquisition section : thanks for including the fact that the annotation process is described in the original article, even though I think that's it could be a bit deepen in the paper, as it presents deep learning models. The annotation are the ground truth and the input of the model(s), it should be very clear what they are.*

AR: We incorporated more detail on data acquisition and format within this section.

Revisions in lines: 301-321

Comment 2.4: Methods

RC: *Method section : I would shorten the first sentence « Our methodology... » to just state « Our methodology entains four analytical steps : first. . . . » because here, some things are well motivated in the intro / background (the computation of embeddings to evaluate BirdNet's transfer learning abilities, the evaluation of individual indentifiability to evaluate how « ecologically-meaningful » is the communication), but some seems to be a bit less obvious (why modifying frequencies ? Why studying SNR ?), and then well motivated in the next paragraphs.*

AR: Thank you for the suggestion, we incorporated this in the text.

Revisions in lines: 323-325

Comment 2.5: Methods A

RC: *Section A : good to have specified how embeddings were computed. I think it would be beneficial to motivate the SVM-head choice (why not choosing a MLP for instance?)*

AR: An SVM is one of the simplest and most interpretable classifiers; high classification accuracy implies that there is a separating hyperplane in the high-dimensional embedding space that can separate most points between the different classes. This approach most directly demonstrates generalizeability of BirdNET embeddings. We added this point in the text.

Revisions in lines: 345-348

Comment 2.6: Methods B

RC: *Section B : l434 « above classification pipeline » l. 434 : which one ? The embeddings + SVM one ? It just lacks a bit of clarity here, as many pieces of interesting info are given. I really like how the noise was taken into account and how you spent time making sure it was not biasing the performances.*

AR: Thank you! We added a clarification here.

Revisions in lines: 362-363

Comment 2.7: Methods C & D

RC: *Section C : no major changes, ok for me.*
Section D : no major changes, ok for me.

AR: Thank you.

Comment 2.8: Results A & B

RC: *Results section :*
Section A : no major changes, ok for me.
Section B : l. 570 « broadly the range most occupied by bird calls », this statement would benefit from a reference I think.

AR: Sure, we added a reference for this statement.

Revisions in lines: 447

Comment 2.9: Results C & D

RC: *Section C : no major changes, OK for me.*
Section D : I really like the fact that detection and classification tasks were led separately, to underlines what whas said

in the intro about the different perceptual spaces. Interesting to see the curves not converging at the same moment. I agree that « the difference between these curves gives the difference between detection and classification », but I would mention that this is true for the model, and not automatically for the animals (and maybe mention that this point is discussed in the discussion section right after)

AR: We added the suggested clarification in several statements within the manuscript, thank you. As the discussion of neural networks and perception was moved to the Background earlier in the manuscript by Reviewer Comment 3.1, we did not add the second point mentioned in the comment.

Revisions in lines: 488, 557-561, Abstract

Comment 2.10: Discussion A

RC: *Discussion section :*
Section A : adding this section was definitely essential for the study, thank you for doing it. l. 694« in the light of the strong parallel » would benefit from references. I am still a bit skeptical about the use of DNN as proxies, and the fact that DNN have proven to be good proxies for human auditory perception does not mean it's gonna work for marine mammal IMO. We do not have the same perceptual space (Uexküll's notion of umwelt). However, the mention of this is interesting, as by automatically detect and classify signal, one could say they are trying to mimick the work of human annotators. I think I would rather draw the parallel here than saying that because DNN suceed in mimicking human auditory perceptions, then it is relevant for marine mammals. This is especially true since signals were transformed (frequency shifted, denoised, etc., which is interesting and well motivated but which pulls them away from « natural » sounds).

AR: Certainly – the line "in light of the strong parallel" was intended to be explained and referenced by the following paragraph. We added additional references at the end of this sentence. While we agree that humans and marine mammals do not have the same perceptual space, it is worth noting the structure of neural networks is not specific to the human brain, but rather to general principles of biological neural processing; the predominant human bias is brought by the human-centric training datasets. We hope to make DNNs more relevant to marine mammals by fine-tuning models on their vocalizations, and added this point in the text.

Revisions in lines: 218, 244-246

Comment 2.11: Discussion B, C, & D

RC: *Section B : I really like the parallel draw between the results presented and the ones that could be found in desnity estimation studies, through physics and propagation models.*
Section C : I really like the closed-set to open-set perspective
Section D : No major changes, ok for me

AR: Thank you!

Reviewer #3

Comment 3.1

RC: *The main concern in the original manuscript, which other reviewers point out as well, is the need for further evidence supporting the use of automatic classification scores as proxies for animal perception. While the authors have addressed this by extending the Discussion, this is a critical point that it should be introduced earlier. I suggest moving these supporting arguments and examples into the Background section, since it would ensure readers are aligned with the methodology from the start, rather than questioning the premise throughout the paper.*

AR: We relocated the discussion of auditory perception to the Background section to better motivate the methodology of the paper. Thank you for this suggestion.

Revisions in lines: 204-253

Comment 3.2

RC: *Additionally, when discussing other deep Learning models (L.282), the authors should cite works comparing pre-trained models used as backbones for downstream tasks. some examples are: Miron, Marius, et al. "What matters for bioacoustic encoding." arXiv preprint arXiv:2508.11845 (2025). Schwinger, Raphael, et al. "Foundation Models for Bioacoustics–a Comparative Review." arXiv preprint arXiv:2508.01277 (2025). Kather, Vincent S.. et al. "Clustering and novel class recognition: evaluating bioacoustic deep learning feature extractors." arXiv preprint arXiv:2504.06710 (2025).*

AR: Thank you for these suggestions! We incorporated these references in the Background section.

Revisions in lines: 144-175

Comment 3.2

RC: *L.154, "Models have shown .. sensitivity to acoustic features..", While I understand the intent here, this phrasing may inadvertently misrepresent our current understanding of what deep learning embeddings actually encode. In bioacoustics, a definitive connection between embeddings and specific acoustic features (such as F0, duration etc.. has not yet been established.*

AR: For sure, we had meant "features" as "characteristics" rather than as specific acoustic measurements. We re-phrased this sentence.

Revisions in lines: 77-78
