# Supplementary material for: Detection, communication, and individual identification with deep audio embeddings: A case study with North Atlantic right whales
Source: PLoS Comput Biol. 2026 Jun 30;22(6):e1013321. doi: 10.1371/journal.pcbi.1013321 (PMC13345461; doi:10.1371/journal.pcbi.1013321)
Supplement: Peer review history

# Peer review: Decision Letter 0

**DOI:** 10.1371/journal.pcbi.1013321.r001

**Authors:** Natalia L. Komarova, Dan Stowell

12 Aug 2025

PCOMPBIOL-D-25-01388

Detection, communication, and individual identification with deep audio embeddings: A case study with North Atlantic right whales

PLOS Computational Biology

Dear Dr. Tolkova,

Thank you for submitting your manuscript to PLOS Computational Biology. After careful consideration, we feel that it has merit but does not fully meet PLOS Computational Biology's publication criteria as it currently stands. Therefore, we invite you to submit a revised version of the manuscript that addresses the points raised during the review process.

Please submit your revised manuscript within 30 days Oct 12 2025 11:59PM. If you will need more time than this to complete your revisions, please reply to this message or contact the journal office at ploscompbiol@plos.org. When you're ready to submit your revision, log on to https://www.editorialmanager.com/pcompbiol/ and select the 'Submissions Needing Revision' folder to locate your manuscript file.

Please include the following items when submitting your revised manuscript:

* A rebuttal letter that responds to each point raised by the editor and reviewer(s). You should upload this letter as a separate file labeled 'Response to Reviewers'. This file does not need to include responses to formatting updates and technical items listed in the 'Journal Requirements' section below.

* A marked-up copy of your manuscript that highlights changes made to the original version. You should upload this as a separate file labeled 'Revised Manuscript with Track Changes'.

* An unmarked version of your revised paper without tracked changes. You should upload this as a separate file labeled 'Manuscript'.

If you would like to make changes to your financial disclosure, competing interests statement, or data availability statement, please make these updates within the submission form at the time of resubmission. Guidelines for resubmitting your figure files are available below the reviewer comments at the end of this letter.

We look forward to receiving your revised manuscript.

Kind regards,

Dan Stowell

Academic Editor

PLOS Computational Biology

Natalia Komarova

Section Editor

PLOS Computational Biology

**Additional Editor Comments:**

The reviewers are in agreement that this paper represents an interesting contribution to the literature, empirically demonstrating a useful point about animal communication in noise. There are many reviewer comments, and all should be addressed in the resubmission. The reviewers are very much in agreement with each other, so I do not need to provide any further guidance - I trust you will find the reviews helpful.

**Journal Requirements:**

If the reviewer comments include a recommendation to cite specific previously published works, please review and evaluate these publications to determine whether they are relevant and should be cited. There is no requirement to cite these works unless the editor has indicated otherwise.

1) We ask that a manuscript source file is provided at Revision. Please upload your manuscript file as a .doc, .docx, .rtf or .tex. If you are providing a .tex file, please upload it under the item type u2018LaTeX Source Fileu2019 and leave your .pdf version as the item type u2018Manuscriptu2019.

2) Please provide an Author Summary. This should appear in your manuscript between the Abstract (if applicable) and the Introduction, and should be 150-200 words long. The aim should be to make your findings accessible to a wide audience that includes both scientists and non-scientists. Sample summaries can be found on our website under Submission Guidelines:

https://journals.plos.org/ploscompbiol/s/submission-guidelines#loc-parts-of-a-submission

3) Please upload all main figures as separate Figure files in .tif or .eps format. For more information about how to convert and format your figure files please see our guidelines:

https://journals.plos.org/ploscompbiol/s/figures

4) Thank you for stating 'All upcall recordings and code to reproduce our analysis is available at https://github.com/avokloti-streamlit/narw-acoustic-identification.' Please note that, though access restrictions are acceptable now, your entire minimal dataset will need to be made freely accessible if your manuscript is accepted for publication. This policy applies to all data except where public deposition would breach compliance with the protocol approved by your research ethics board. If you are unable to adhere to our open data policy, please kindly revise your statement to explain your reasoning and we will seek the editor's input on an exemption.

**Reviewers' comments:**

Reviewer's Responses to Questions

**Comments to the Authors:**

**Please note here if the review is uploaded as an attachment.**

Reviewer #1: This work presents a novel perspective on using deep audio embeddings for conservation purposes, by proposing a framework to estimate the communication space of a species, going beyond detection. This shift in paradigm is an important and necessary contribution to the field. Also, the efforts made to eliminate the background noise as a confounding factor is commendable, and rare enough to be highly appreciated. What mainly lacks still is the completion of the introduction regarding:

- why would the individual identity information be of importance to the NARW

- prior works of modelling intelligibility with deep audio embeddings

- other existing bioacoustic deep audio embedders

Below are specific comments:

"Models trained on bioacoustic data..."

Since models is plural here, could you mention the other networks that have been proposed for such purposes? I am thinking of Hagiwara 2023 and Best 2022 for instance. Regrettably, I have not seen a comparison of Birdnet and these two models (there might be more), despite they all claim to serve the same purpose of generic deep embddings for bioacoustic data. Comparing them for this use case would be great, but you might deem it to be out of scope for this study. Since deep audio embeddings are central to this paper, I believe an introduction on the varying models that exist and how they differ (e.g. training procedure, training data) is necessary.

"AVES: Animal Vocalization Encoder Based on Self-Supervision" Hagiwara 2023

"Deep audio embeddings for vocalization clustering" Best et al. 2022

"In this light, machine learning could also provide a new opportunity to quantify information transfer within animal vocalizations, filling a key gap in the study of communication."

The connection between previous findings on the capability of deep embeddings to discriminate between calls, species or individuals, and the fact that they can model perception abilities in non-humans seems too weak. After a quick search, it seems this question has been already studied for birds (Morfi et al. 2021) and for humans (Haro et al. 2020, Best et al. 2024). Specifically for the research question of this paper, the field of speech intelligibility prediction seems appropriate to explore/refer to. The previous argumentation gives a great introduction, which just needs to be completed with a review of literature on modelling sound perception with deep learning models.

"Deep perceptual embeddings for unlabelled animal sound events" Morfi et al. 2021

"Deep Neural Network Model of Hearing-Impaired Speech-in-Noise Perception" Haro et al. 2020

“Transfer learning from Whisper for microscopic intelligibility prediction,” Best et al. 2024

"As all whales, NARW rely on sound for communication"

perhaps 'repertoire' is more appropriate here than 'vocabulary'?

Besides the mother-calf bond maintenance mentioned later, are there other functions to this communication that make it vital for the whales?

"The impacts of acoustic masking have wide-ranging impacts on a social species"

Maybe remove "The impacts of"?

"Recordings of upcalls therefore provide..."

Could you discuss why/how individual identity could be important to the whales? It seems crucial for the claims of this paper. If a higher SNR is necessary for individual ID as compared to call type discrimination, and if only the call type information actually matters to the whales, the communication space would be underestimated.

Depending on the hypotheses on the importance of individual ID, could you make reference to the framework of Dooling et al. to classify in which category do you place the space in which individual ID is possible?

"quantify communication"

This seems odd, could you reformulate?

"adapting to lower-frequency data"

adapting what?

"...within prior estimates, ..."

Maybe replace estimates with findings?

"standardized to a length of 3 seconds by zeropadding."

Why is this necessary? Would it not be possible to simply extract 3 seconds of signal around each call? This seems particularly important if the duration of the call carries individual information as stated later. If calls are pre-cut manually and zero-padded, it would be a strong help to the following pipeline and has to be mentioned.

Could you specify the median and std for number of calls per individual?

If I understand correctly, all calls from a given individual were collected with one tag. If deemed reasonable, please discuss the possibility that the variation between upcalls could arise from behavioral variations instead of individual signatures (e.g. if one individual was more stressed than another during the tag recording, this could generate upsweep shape differences in this dataset which would not appear in recordings from another context).

"In fact, BirdNET embeddings have yielded higher classification accuracies for downstream bioacoustic tasks..."

Again here, it would seem fair to mention other models proposed for embedding bioacoustic data.

"BirdNET Analyzer v1.5.1"

Could there be a citation to ease finding this software?

"vectors of 1024 features for each audio sample"

Does this imply a mean pooling of the 3 seconds excerpts? This is crucial and should be detailed and discussed.

"with these acoustic features"

It seems odd to describe audio embeddings as acoustic features, since the term is often used to describe formalised acoustic measurements.

"we could infer that tag-specific audio characteristics were a significant driver of the variation in upcall samples."

This sentence seems more linked to section III C, I don't understand the connection to the rest of the paragraph.

"BirdNET embeddings were learning"

It seems that learning is not accurately used here.

"classification pipeline this additional"

Missing 'to'?

Could Figure 1 be mentioned already in the methods section to help readers understand the procedures described?

In Fig.3 B, I understand that showing upcall spectrograms can help interpret the figure but it also makes it difficult to see clusters with overlapping snipets. I believe individual positions in the low dimensional space here is more important than visualising patterns in the snipets which is hardly doable. Could you consider switching to points, maybe with some transparency to avoid masking in case of overlaps?

"we selected de-noised upcall samples for the best performing denoising threshold"

Isn't the best performing denoising threshold also a threshold at which classification is possible from background noise alone?

Section III E

Using more realistic noise instead of white noise (boat sound or background noise from the DTAGS for instance) would improve the strength of the analysis here.

"these results suggest robustness of BirdNET to new signals"

The results discussed here show the impact domain knowledge can have in adapting signals for a given model, rather than the direct robustness of BirdNet.

"Accuracies for the upcall dataset are shown in solid lines, ..."

IMO this belongs more to the legend of the figure.

Could you add the information of performance for the upcall and the noise datasets in the case of no spectral substraction? Ideally in Figure 3 left, or at least in the text?

"correlated factors"

Would confounding factors be more appropriate here?

Are the embeddings in Figure 3 B from the original sampling rate or the sped up (most accurate) version?

"the smaller separate cluster at the edge of the figure"

It is unclear to me to what this corresponds to.

"However, this task is substantially more challenging..."

I do not agree with this statement. Although I agree applying abundance estimate to tag recordings is conceptually absurd, the dataset used in this study could be used to evaluate audio embeddings in an open-set setting, simply by switching from training an SVM and measuring accuracy to applying a clustering algorithm and measuring the NMI or the number of missed / double counted individuals. Augmenting the data with SNR variations, we could evaluate audio embeddings for an open-set AIID task and inform on their applicability to PAM recordings.

"However, a number of external factors may affect acoustic signatures,"

In reference to the previous comment regarding this, can you discuss these factors not only for the potential collection of PAM AIID data, but also for the dataset used here?

The first paragraph of Section V B is difficult to understand and hard to put in context.

Could you add a quick summary to the main findings regarding the gap between detection and recognition and the corresponding "communication space" reduction in the conclusion and abstract?

Reviewer #2: review uploeaded as an attachment

Reviewer #3: This work focuses on how to measure the impact that human-made noise has on the communication ability of vocal animals. Importantly, the authors describe assessing communication ability as more complex than simply detecting signals, and use the problem of individual identification in North Atlantic Right Whales (NARW) to exemplify this crucial distinction.

In short, deep learning models are used to encode the calls, and identification accuracy is measured under several levels of added noise. The authors show that basing impact measures purely on detection performance leads to a significant underestimation, and that deep learning models can capture the complex particularities of the signals needed for effective information exchange.

Overall, the manuscript is clearly written and describes a thoroughly considered project.

I find it detracts somewhat that the authors only address the clear difference between NARW ID recognition ability and audio embeddings at the end of the manuscript.

While they mention that using audio embeddings as “a proxy for the perceptive capabilities of NARW” is an “imperfect comparison,” the justification could be improved and advantages highlighted.

Finally, a reflection on how this assessment could be applied to other forms of communication and species would help move beyond the specific case study and describe a more generalised process for evaluating the impact of human-made noise.

smaller comments below:

[Abstract] "communication space -the area.." --> instead of area, should it be space?

[INTRO p.3] "we aim to quantify communication.." --> unclear or too vague.

[III-A] " vectors of 1024 features for each audio sample" --> not clear what is an audio sample here, 3 seconds of audio? is the final embedding averaged over time?

[III-B] "low sample size..." --> dataset size?

[IV-D] "intermediate values..", not clear what values means here, accuracy values?

**********

**Have the authors made all data and (if applicable) computational code underlying the findings in their manuscript fully available?**

The PLOS Data policy requires authors to make all data and code underlying the findings described in their manuscript fully available without restriction, with rare exception (please refer to the Data Availability Statement in the manuscript PDF file). The data and code should be provided as part of the manuscript or its supporting information, or deposited to a public repository. For example, in addition to summary statistics, the data points behind means, medians and variance measures should be available. If there are restrictions on publicly sharing data or code —e.g. participant privacy or use of data from a third party—those must be specified.

Reviewer #1: **No:**  The link in the data availability statement is broken

Reviewer #2: **No:**  The github page provided in the manuscript yields a 404 error. It may be unavailable during the review and then will be public once the revew process is over.

Reviewer #3: Yes

**********

PLOS authors have the option to publish the peer review history of their article (what does this mean?). If published, this will include your full peer review and any attached files.

If you choose “no”, your identity will remain anonymous but your review may still be made public.

**Do you want your identity to be public for this peer review?**  For information about this choice, including consent withdrawal, please see our Privacy Policy.

Reviewer #1: **Yes:**  Paul Best

Reviewer #2: No

Reviewer #3: No

[NOTE: If reviewer comments were submitted as an attachment file, they will be attached to this email and accessible via the submission site. Please log into your account, locate the manuscript record, and check for the action link "View Attachments". If this link does not appear, there are no attachment files.]

**Figure resubmission:**

While revising your submission, please upload your figure files to the Preflight Analysis and Conversion Engine (PACE) digital diagnostic tool, https://pacev2.apexcovantage.com/. PACE helps ensure that figures meet PLOS requirements. To use PACE, you must first register as a user. Registration is free. Then, login and navigate to the UPLOAD tab, where you will find detailed instructions on how to use the tool. If you encounter any issues or have any questions when using PACE, please email PLOS at figures@plos.org. Please note that Supporting Information files do not need this step. If there are other versions of figure files still present in your submission file inventory at resubmission, please replace them with the PACE-processed versions.

**Reproducibility:**

To enhance the reproducibility of your results, we recommend that authors of applicable studies deposit laboratory protocols in protocols.io, where a protocol can be assigned its own identifier (DOI) such that it can be cited independently in the future. Additionally, PLOS ONE offers an option to publish peer-reviewed clinical study protocols. Read more information on sharing protocols at https://plos.org/protocols?utm_medium=editorial-email&utm_source=authorletters&utm_campaign=protocols

Attachment Submitted filename: review.pdf

# Author comment: Author response to Decision Letter 1

**DOI:** 10.1371/journal.pcbi.1013321.r002

23 Jan 2026

Attachment Submitted filename: NARW_ID__Response.pdf

# Peer review: Decision Letter 1

**DOI:** 10.1371/journal.pcbi.1013321.r003

**Authors:** Natalia L. Komarova, Dan Stowell

18 Feb 2026

PCOMPBIOL-D-25-01388R1

Detection, communication, and individual identification with deep audio embeddings: A case study with North Atlantic right whales

PLOS Computational Biology

Dear Dr. Tolkova,

Thank you for submitting your manuscript to PLOS Computational Biology. After careful consideration, we feel that it has merit but does not fully meet PLOS Computational Biology's publication criteria as it currently stands. Therefore, we invite you to submit a revised version of the manuscript that addresses the points raised during the review process.

Please submit your revised manuscript by Apr 20 2026 11:59PM. If you will need more time than this to complete your revisions, please reply to this message or contact the journal office at ploscompbiol@plos.org. When you're ready to submit your revision, log on to https://www.editorialmanager.com/pcompbiol/ and select the 'Submissions Needing Revision' folder to locate your manuscript file.

Please include the following items when submitting your revised manuscript:

* A letter that responds to each point raised by the editor and reviewer(s). You should upload this letter as a separate file labeled 'Response to Reviewers'. This file does not need to include responses to formatting updates and technical items listed in the 'Journal Requirements' section below.

* A marked-up copy of your manuscript that highlights changes made to the original version. You should upload this as a separate file labeled 'Revised Manuscript with Track Changes'.

* An unmarked version of your revised paper without tracked changes. You should upload this as a separate file labeled 'Manuscript'.

If you would like to make changes to your financial disclosure, competing interests statement, or data availability statement, please make these updates within the submission form at the time of resubmission. Guidelines for resubmitting your figure files are available below the reviewer comments at the end of this letter.

We look forward to receiving your revised manuscript.

Kind regards,

Dan Stowell

Academic Editor

PLOS Computational Biology

Natalia Komarova

Section Editor

PLOS Computational Biology

**Additional Editor Comments:**

Thank you for your work on the resubmission. The 3 reviewers are in broad agreement that the improvements that have been made are good. However, all 3 reviewers also raised minor issues that should still be addressed before proceeding. Please do attend to these and resubmit.

**Journal Requirements:**

Please upload all main figures as separate Figure files in .tif or .eps format. For more information about how to convert and format your figure files please see our guidelines:

https://journals.plos.org/ploscompbiol/s/figures

**Reviewers' comments:**

Reviewer's Responses to Questions

**Comments to the Authors:**

**Please note here if the review is uploaded as an attachment.**

Reviewer #1: - comments 1.15 and 3.5: Two reviewers have asked for clarification on the pooling of embeddings within 3 second excerpts, but the information was still not added. It would be great if authors could give more details on the procedure of embeddings generation, without asking readers to go through the command-line interface documentation.

- comment 1.22: Thanks for clarifying, could you still add the specific denoising threshold used?

- The reference is missing for the first two mentions of clark et al (2009)

- "25 times greater then" should this be "greater than"?

- "an 7 dB difference" should this be "a 7 dB difference"?

Reviewer #2: Review uploaded as an attachment.

Reviewer #3: Thank you for revising the manuscript.

The main concern in the original manuscript, which other reviewers point out as well, is the need for further evidence supporting the use of automatic classification scores as proxies for animal perception. While the authors have addressed this by extending the Discussion, this is a critical point that it should be introduced earlier. I suggest moving these supporting arguments and examples into the Background section, since it would ensure readers are aligned with the methodology from the start, rather than questioning the premise throughout the paper.

Additionally, when discussing other deep Learning models (L.282), the authors should cite works comparing pre-trained models used as backbones for downstream tasks. some examples are: Miron, Marius, et al. "What matters for bioacoustic encoding." arXiv preprint arXiv:2508.11845 (2025).

Schwinger, Raphael, et al. "Foundation Models for Bioacoustics--a Comparative Review." arXiv preprint arXiv:2508.01277 (2025).

Kather, Vincent S.. et al. "Clustering and novel class recognition: evaluating bioacoustic deep learning feature extractors." arXiv preprint arXiv:2504.06710 (2025).

L.154, "Models have shown .. sensitivity to acoustic features..", While I understand the intent here, this phrasing may inadvertently misrepresent our current understanding of what deep learning embeddings actually encode. In bioacoustics, a definitive connection between embeddings and specific acoustic features ( such as F0, duration etc.. has not yet been established.

**********

**Have the authors made all data and (if applicable) computational code underlying the findings in their manuscript fully available?**

The PLOS Data policy requires authors to make all data and code underlying the findings described in their manuscript fully available without restriction, with rare exception (please refer to the Data Availability Statement in the manuscript PDF file). The data and code should be provided as part of the manuscript or its supporting information, or deposited to a public repository. For example, in addition to summary statistics, the data points behind means, medians and variance measures should be available. If there are restrictions on publicly sharing data or code —e.g. participant privacy or use of data from a third party—those must be specified.

Reviewer #1: Yes

Reviewer #2: **No:**

Reviewer #3: Yes

**********

PLOS authors have the option to publish the peer review history of their article (what does this mean?). If published, this will include your full peer review and any attached files.

If you choose “no”, your identity will remain anonymous but your review may still be made public.

**Do you want your identity to be public for this peer review?**  For information about this choice, including consent withdrawal, please see our Privacy Policy.

Reviewer #1: **Yes:**  Paul Best

Reviewer #2: No

Reviewer #3: **Yes:**  Ines Nolasco

[NOTE: If reviewer comments were submitted as an attachment file, they will be attached to this email and accessible via the submission site. Please log into your account, locate the manuscript record, and check for the action link "View Attachments". If this link does not appear, there are no attachment files.]

**Figure resubmission:**

-->While revising your submission, we strongly recommend that you use PLOS’s NAAS tool (https://ngplosjournals.pagemajik.ai/artanalysis) to test your figure files. NAAS can convert your figure files to the TIFF file type and meet basic requirements (such as print size, resolution), or provide you with a report on issues that do not meet our requirements and that NAAS cannot fix.

After uploading your figures to PLOS’s NAAS tool - https://ngplosjournals.pagemajik.ai/artanalysis, NAAS will process the files provided and display the results in the "Uploaded Files" section of the page as the processing is complete. If the uploaded figures meet our requirements (or NAAS is able to fix the files to meet our requirements), the figure will be marked as "fixed" above. If NAAS is unable to fix the files, a red "failed" label will appear above. When NAAS has confirmed that the figure files meet our requirements, please download the file via the download option, and include these NAAS processed figure files when submitting your revised manuscript.

**Reproducibility:**

To enhance the reproducibility of your results, we recommend that authors of applicable studies deposit laboratory protocols in protocols.io, where a protocol can be assigned its own identifier (DOI) such that it can be cited independently in the future. Additionally, PLOS ONE offers an option to publish peer-reviewed clinical study protocols. Read more information on sharing protocols at https://plos.org/protocols?utm_medium=editorial-email&utm_source=authorletters&utm_campaign=protocols

Attachment Submitted filename: review2.pdf

# Author comment: Author response to Decision Letter 2

**DOI:** 10.1371/journal.pcbi.1013321.r004

30 Apr 2026

Attachment Submitted filename: NARW_ID__Response_to_Reviewers__Revision2.pdf

# Editor report: Decision Letter 2

**DOI:** 10.1371/journal.pcbi.1013321.r005

**Authors:** Natalia L. Komarova, Dan Stowell

18 May 2026

Dear Dr. Tolkova,

We are pleased to inform you that your manuscript 'Detection, communication, and individual identification with deep audio embeddings: A case study with North Atlantic right whales' has been provisionally accepted for publication in PLOS Computational Biology.

Before your manuscript can be formally accepted you will need to complete some formatting changes, which you will receive in a follow up email. A member of our team will be in touch with a set of requests.

Please note that your manuscript will not be scheduled for publication until you have made the required changes, so a swift response is appreciated.

IMPORTANT: The editorial review process is now complete. PLOS will only permit corrections to spelling, formatting or significant scientific errors from this point onwards. Requests for major changes, or any which affect the scientific understanding of your work, will cause delays to the publication date of your manuscript.

Should you, your institution's press office or the journal office choose to press release your paper, you will automatically be opted out of early publication. We ask that you notify us now if you or your institution is planning to press release the article. All press must be co-ordinated with PLOS.

Thank you again for supporting Open Access publishing; we are looking forward to publishing your work in PLOS Computational Biology.

Best regards,

Dan Stowell

Academic Editor

PLOS Computational Biology

Natalia Komarova

Section Editor

PLOS Computational Biology

***********************************************************

Thank you for revising the manuscript according to the reviewers' latest feedback. The organisation of the manuscript is improved, in particular with the background section which considers the methodological motivations.

(One small note: Morfi et al (2021) studied zebra finch, not chaffinch.)

# Editor report: Acceptance letter

**DOI:** 10.1371/journal.pcbi.1013321.r006

**Authors:** Natalia L. Komarova, Dan Stowell

PCOMPBIOL-D-25-01388R2

Detection, communication, and individual identification with deep audio embeddings: A case study with North Atlantic right whales

Dear Dr Tolkova,

I am pleased to inform you that your manuscript has been formally accepted for publication in PLOS Computational Biology. Your manuscript is now with our production department and you will be notified of the publication date in due course.

The corresponding author will soon be receiving a typeset proof for review, to ensure errors have not been introduced during production. Please review the PDF proof of your manuscript carefully, as this is the last chance to correct any errors. Please note that major changes, or those which affect the scientific understanding of the work, will likely cause delays to the publication date of your manuscript.

Soon after your final files are uploaded, unless you have opted out, the early version of your manuscript will be published online. The date of the early version will be your article's publication date. The final article will be published to the same URL, and all versions of the paper will be accessible to readers.

For Research, Software, and Methods articles, you will receive an invoice from PLOS for your publication fee after your manuscript has reached the completed accept phase. If you receive an email requesting payment before acceptance or for any other service, this may be a phishing scheme. Learn how to identify phishing emails and protect your accounts at https://explore.plos.org/phishing.

Thank you again for supporting PLOS Computational Biology and open-access publishing. We are looking forward to publishing your work!

With kind regards,

Anitha Samidurai

PLOS Computational Biology | Carlyle House, Carlyle Road, Cambridge CB4 3DN | United Kingdom ploscompbiol@plos.org | Phone +44 (0) 1223-442824 | ploscompbiol.org | @PLOSCompBiol
